# Photoplethysmography-based HRV analysis and machine learning for real-time stress quantification in mental health applications

**DOI:** 10.1063/5.0256590

**Published:** 2025-04-03

**Authors:** Ying-Ying Tsai, Yu-Jie Chen, Yen-Feng Lin, Fan-Chi Hsiao, Ching-Han Hsu, Lun-De Liao

**Affiliations:** 1 Institute of Biomedical Engineering and Nanomedicine, National Health Research Institutes, 35, Keyan Road, Zhunan Town, Miaoli County 350, Taiwan; 2 Department of Biomedical Engineering and Environmental Sciences, National Tsing-Hua University, Hsinchu, Taiwan; 3 Center for Neuropsychiatric Research, National Health Research Institutes, 35, Keyan Road, Zhunan Town, Miaoli County 350, Taiwan; 4 Department of Counseling, Clinical and Industrial/Organizational Psychology, Ming Chuan University, Taoyuan, Taiwan

## Abstract

Prolonged exposure to high-stress environments can lead to mental illnesses such as anxiety disorders, depression, and posttraumatic stress disorder. Here, a wearable device utilizing photoplethysmography (PPG) technology is developed to noninvasively measure physiological signals and analyze heart rate variability (HRV) parameters. Traditional normative HRV databases typically do not account for responses induced by specific stressors such as cognitive tasks. Therefore, machine learning is used to build a more dynamic stress assessment model. Machine learning can capture complex nonlinear relationships among HRV parameters during stress-inducing tasks, adapts to individual stress response variations, and provides real-time stress level predictions. Furthermore, machine learning models can integrate temporal patterns in HRV data to achieve nuanced stress level assessment. This study examines the feasibility of PPG signals and validates the developed stress model. The RR intervals derived from PPG signals were highly positively correlated with those from electrocardiography signals (correlation coefficient = 0.9920, R-squared = 0.9837); this confirms the usability of PPG signals for HRV analysis. The stress model is constructed via the open-source Swell dataset. In the experiments, participants complete the Depression Anxiety Stress Scales-21-Chinese (DASS-21-C) questionnaire to quantify levels of depression, anxiety, and stress over a week. Baseline and stress-state PPG data are collected, converted into HRV values, and input into the model for stress quantification. The Stroop test is used to elicit stress responses. After the experiment, the DASS-21-C stress scores were compared with the model's baseline, stress state, and combined scores. The highest correlation was observed between the model's baseline score and the DASS-21-C stress score (correlation coefficient = 0.92, R-squared =  0.8457), supporting the model's psychological significance in quantifying everyday stress. HRV parameter changes across experimental phases are discussed as well as sex differences in stress responses. In the future, this device may be applied in clinical scenarios for further validation and could be integrated with additional physiological indicators for broader application in daily health management and stress warning systems.

## I. INTRODUCTION

In modern society, people frequently encounter various sources of stress, including work-related, financial, social, and familial stress.^1^ In highly competitive work environments, high demands and time pressures often lead to both physical and mental exhaustion. Moreover, the rising cost of living and the widening income gap exacerbate financial stress.^2^ Social pressures, including the need to meet others' expectations and perform well, frequently lead to anxiety and self-doubt.^3^ Family dynamics and expectations also contribute significantly to stress, often impacting individuals' emotional and psychological well-being.^4^ The fast pace and high-pressure environment of modern society have introduced unprecedented challenges that may result in or exacerbate mental health issues. Consequently, individuals are increasingly susceptible to the effects of stress, which can manifest as a range of mental health disorders,^5,6^ including anxiety disorders, depression, posttraumatic stress disorder (PTSD), and insomnia.^7^ Long-term exposure to stress can cause emotional disturbances such as anxiety, depression, and insomnia, all of which significantly impair both physical and mental health.^8,9^

In stressful situations, particularly for individuals with preexisting mental health conditions, the body's acute stress response is activated, which is primarily governed by the sympathetic nervous system. This system initiates physiological changes across the cardiovascular, respiratory, muscular, and metabolic systems to cope with stress.^10^ The heart rate and blood pressure increase to supply more oxygen and energy to the muscles and brain.^11^ The respiratory rate increases, facilitating increased oxygen intake.^12^ Muscle contractions intensify to prepare the body for action,^13^ and the metabolic system releases glucose into the bloodstream, providing additional energy.^14^ The adrenal glands, which are part of the sympathetic nervous system, release adrenaline, a key hormone in the stress response.^15^ Adrenaline accelerates heart rate, increases blood pressure, dilates airways, and increases energy metabolism, enabling individuals to respond effectively to stressors.^16,17^

The growing awareness of mental health issues has highlighted the importance of both mental and physical well-being.^18,19^ Many individuals now seek professional psychological and medical support when faced with mental health challenges. Psychologists and therapists offer a range of therapeutic interventions,^20^ guiding individuals in managing stress and adopting healthier coping strategies.^21^ In therapy, confronting stressors is often essential to the treatment process, as it helps patients address the root causes of stress.^22^ However, patients may experience significant stress fluctuations during therapy, necessitating professional guidance to prevent symptoms from worsening.^23^ Currently, stress levels are typically assessed on the basis of the therapist's subjective judgment.^24^

Some works have considered the relationship between psychological stress and physiological signals. Xia *et al.* conducted a mental arithmetic test with 22 participants and collected electroencephalography (EEG) and electrocardiography (ECG) data. They applied principal component analysis (PCA) for feature extraction and used a support vector machine (SVM) classifier, achieving an accuracy of 79.54% in classifying no stress, mild stress, and severe stress.^25^ Kurniawan *et al.* employed various stress induction methods, such as the Stroop color-word test (Stroop test), the Trier social stress test (TSST), and the Trier mental challenge test (TMCT), on 10 participants and measured their vocal signals and electrodermal activity (EDA). They used a decision tree classifier to determine stress levels, achieving an accuracy of 92.60%.^26^ These studies illustrate the feasibility of using physiological parameters for stress evaluation. However, there are two challenges. First, the use of multiple physiological parameters for stress assessment may complicate home-based monitoring, as it requires costly equipment and technical expertise.^27,28^ Second, traditional stress assessments based solely on the intensity of stress stimuli lack psychological context.^29^ This study addresses these challenges by focusing on a single physiological signal device for effective stress evaluation and defining stress levels through psychological questionnaires.

The aim of this work is to develop an easy-to-wear device that quantifies stress levels,^30^ providing psychologists with an additional tool during treatment. The device uses photoplethysmography (PPG) technology,^31^ a noninvasive method for measuring physiological signals.^32–34^ PPG works by applying infrared light to peripheral tissues, in which the blood volume changes with each heartbeat. The blood absorbs some of the infrared light, and a photodetector measures the remaining light intensity.^35^ By analyzing these light fluctuations, the heart rate is calculated, and the resulting signals are used to analyze heart rate variability (HRV) parameters.^36,37^ Previous studies have explored the relationship between HRV and stress. For example, Koldijk *et al.* analyzed 34 HRV parameters, correlated them with stress levels in an experimental setting, and published the dataset.^38^ The present study uses a decision tree model trained on this dataset to establish an HRV–stress model and validates it experimentally. The participants completed the Depression Anxiety Stress Scales-21-Chinese (DASS-21-C), a psychological tool for assessing stress levels.^39^ Many existing studies focus solely on physiological signals for stress evaluation without incorporating psychological assessments, and this may decrease validity. An innovation of this study is that it integrates the DASS-21-C to establish the psychological relevance of the model. During the experiment, participants' HRV data were collected under resting and stress conditions. The Stroop test, a well-established psychological tool, was used to induce stress responses.^40^ The experiment involved two stress levels designed to assess the effectiveness of the model.

## II. RESULTS

### A. Evaluation of the correlation between the PPG signals and the self-developed device results

**FIG. 1.**
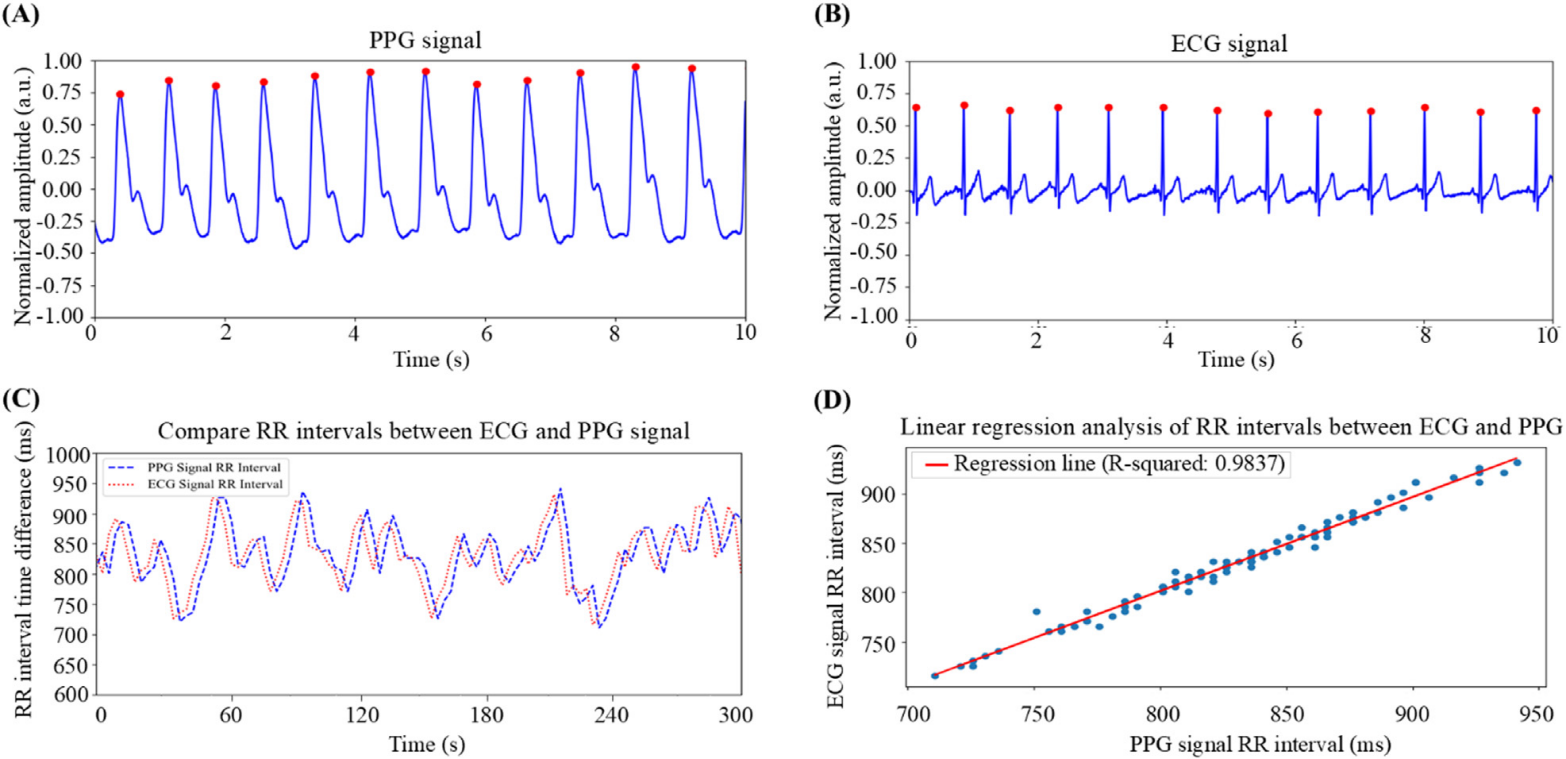
Correlation analysis of RR intervals between PPG and ECG signals. (a) PPG signal graph: This chart displays the raw PPG signal captured via the MAX86150 sensor. The waveform obtained after signal processing is shown, with the peak coordinates representing the heartbeats marked on the peaks of the waveform. (b) ECG signal graph: This graph presents the ECG signal sampled via the MAX86150 sensor. Similar to the PPG signal, the processed ECG waveform is displayed, and the peak coordinates of each heartbeat are marked. (c) RR interval analysis: This panel compares the peak coordinates of the PPG signal from (a) with those of the ECG signal from (b), generating RR interval graphs for both. RR intervals represent the time intervals between consecutive heartbeats and are critical for assessing the rhythm and regularity of the heart. (d) Linear regression correlation analysis: This analysis reveals a strong positive correlation between the RR intervals derived from the PPG and ECG signals, with an R-squared value of 0.9837. This result indicates that RR intervals obtained from PPG signals can effectively replace those obtained from ECG signals to yield similar heart rate information accuracy.

This study first validated the feasibility of using PPG signals for calculating HRV. As shown in Fig. 1, both the PPG and ECG signals were measured simultaneously via a MAX86150 device. After five minutes of measurement, the signals were processed. Both signals were normalized to a range of −1 to 1, and the peaks were identified. The PPG results are shown in Fig. 1(a), and the ECG results are shown in Fig. 1(b). In both figures, the peaks corresponding to heartbeats are clearly visible. By marking each peak, the time between heartbeats, or the RR interval, was calculated. The RR interval derived from the PPG signal was 832.24 ± 56.16 ms on average, whereas that derived from the ECG signal was 848.54 ± 124.62 ms on average. Figure 1(c) shows the RR intervals from the ECG and PPG signals plotted together, revealing similar trends. A linear regression analysis, shown in Fig. 1(d), yielded a correlation coefficient of 0.9918 and an R-squared value of 0.9837, indicating that the RR intervals from the PPG signals are highly correlated with those from the ECG signals. This finding confirms that PPG signals can be reliably used to calculate HRV parameters.

**FIG. 2.**
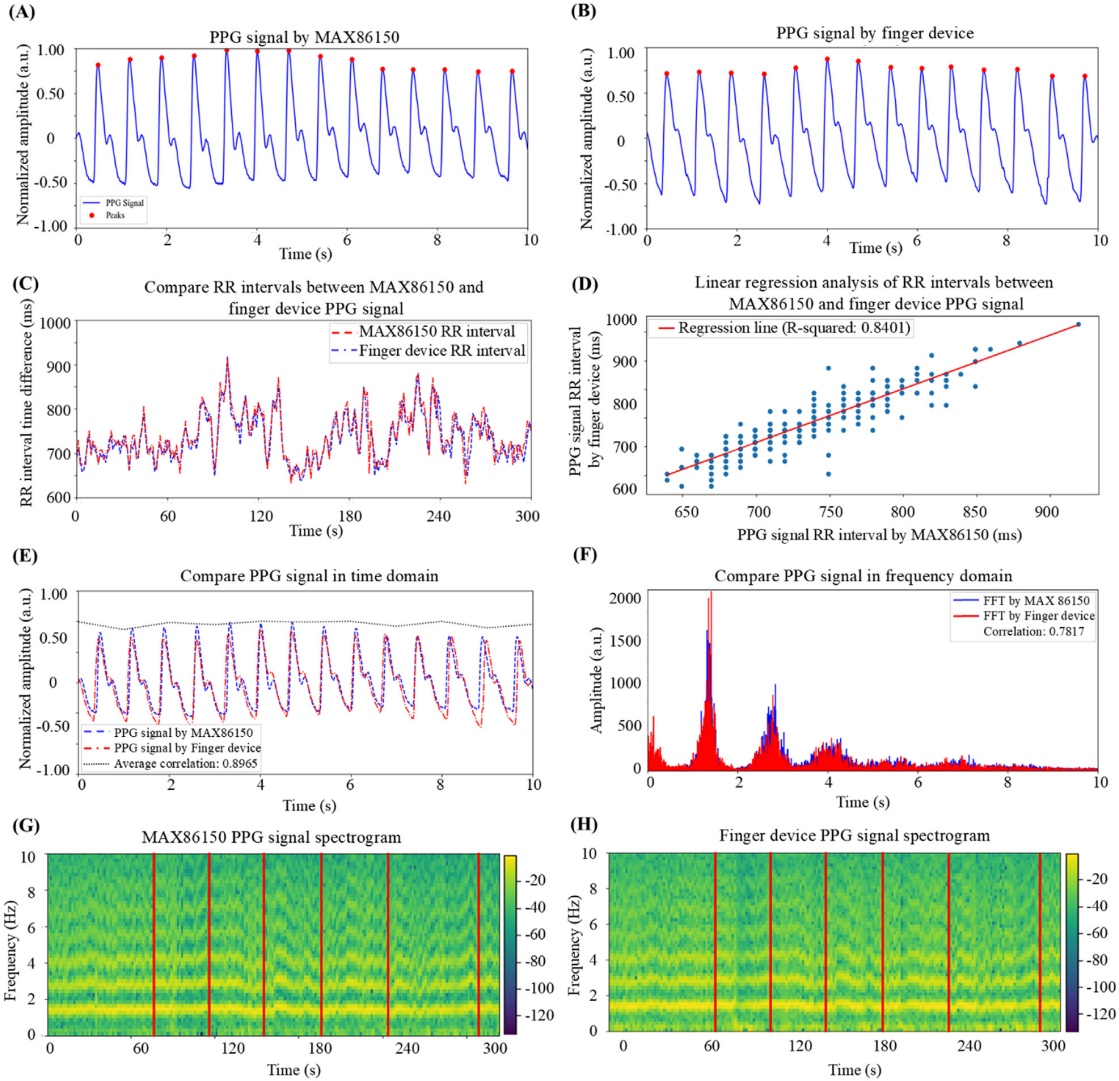
Correlation analysis of the PPG signals between the self-developed wearable device and the MAX86150. (a) MAX86150 PPG signal waveform: This chart shows the PPG signal measured via the MAX86150 sensor. After signal processing, the peaks representing each heartbeat are detected and marked. (b) PPG signal waveform of the self-developed device: This graph presents the PPG signal measured via the self-developed wearable device, with signal processing applied to detect and mark the heartbeat peaks. (c) RR interval analysis: The peak points from (a) and (b) are used to calculate RR intervals for both the MAX86150 and the self-developed device. The resulting RR interval graphs are plotted side by side for comparison. (d) Linear regression correlation analysis: A correlation analysis between the RR intervals derived from the MAX86150 and the self-developed device shows an R-squared value of 0.8401, indicating a strong positive correlation. This demonstrates that the RR intervals obtained from the self-developed device are comparable to those from the MAX86150 PPG signal. (e) Overlay correlation analysis of the PPG signals: The PPG signals from the MAX86150 and the self-developed device are overlaid for comparison. A Pearson correlation analysis, performed using groups of three consecutive sampling points, yields an average correlation coefficient of 0.8966, indicating a strong positive correlation between the two devices. (f) Spectral overlay correlation analysis: The original signals from both devices are transformed via fast Fourier transform (FFT) to produce power spectral density plots. An overlay comparison of the frequency domain results in a correlation coefficient of 0.7817, indicating a high positive correlation between the two devices in terms of frequency components. (g) Time–frequency analysis of MAX86150 PPG signals: A time–frequency analysis of the original PPG signal from the MAX86150 shows the signal's stability over the entire measurement period. (h) Time–frequency analysis of PPG signals from the self-developed device: A similar time–frequency analysis is performed for the NHRI development device's PPG signal. Compared with (g), the amplitude and temporal variations of the PPG signals from both devices are similar, confirming the stability and comparability of the signals measured by the MAX86150 and the self-developed device.

Next, this study developed a wearable device based on PPG. After development, the MAX86150 device and the self-developed device were used simultaneously to measure PPG signals at the fingertip for five minutes. After measurement, the signals were processed with high-pass filtering and normalization, and the peaks were marked to calculate the RR intervals. Figure 2(a) shows the processed PPG signals from the MAX86150, and Fig. 2(b) shows the PPG signals from the self-developed device. After the peaks were marked, the RR intervals were calculated for both devices. Figure 2(c) shows the overlapping plots of the RR intervals from both devices. A linear regression analysis, shown in Fig. 2(d), yielded an R-squared value of 0.8401, indicating that the RR intervals calculated by the self-developed device are highly correlated with those from the MAX86150. To further evaluate the feasibility of the self-developed PPG device, the time-domain and frequency-domain characteristics were analyzed. Figure 2(e) shows a time-domain comparison of the PPG signals from the self-developed device and the MAX86150, revealing high similarity in the waveforms. To quantify the correlation, the Pearson correlation coefficient was calculated for every 10 data points, resulting in an average correlation value of 0.8966, confirming that the signals from the self-developed device are highly correlated with those from the MAX86150 in the time domain. For the frequency-domain analysis, the signals were first subjected to a fast Fourier transform (FFT) to obtain the frequency spectrum for signal composition analysis. As shown in Fig. 2(f), the correlation coefficient between the two frequency spectra was 0.7817, indicating high correlation between the signals from the two devices in the frequency domain. The difference in correlation strength between time-domain (R^2^ = 0.8401) and frequency-domain (R^2^ = 0.7817) analyses warrants further discussion. The time-domain correlation is particularly significant, as RR intervals, the fundamental basis for all HRV calculations, are derived directly from time-domain signals. The strong correlation in time-domain measurements (R^2^ = 0.8401), therefore, provides crucial validation of the device's ability to accurately capture these essential RR intervals. The slightly lower correlation in frequency-domain analysis can be attributed to several factors. First, frequency-domain parameters are computed through transformations of the original time-series data, and this transformation process can amplify minor differences in the original signals. Second, frequency-domain parameters are inherently more sensitive to subtle variations in signal quality and sampling precision. Minor differences in signal acquisition between the two devices, such as variations in sampling rate stability and motion artifacts, have a more pronounced effect on frequency components. Despite these technical considerations, both correlation values remain within acceptable ranges for physiological measurements, and more importantly, the relative changes in both time and frequency domain parameters during stress responses maintain consistent patterns, supporting the reliability of the device for stress monitoring applications.

Finally, a short-term Fourier transform (STFT) was used for time–frequency analysis, allowing variations in the frequency components over time to be observed. As shown in Figs. 2(g) and 2(h), the time–frequency plots for the two devices indicate significant correlations, further confirming the consistency between the self-developed device and the MAX86150. In summary, through time-domain, frequency-domain, and time-frequency analyses, the self-developed device was shown to effectively capture PPG signals that have a high degree of correlation with those measured by the MAX86150. These results demonstrate the feasibility and reliability of using a self-developed device for HRV parameter calculation, providing strong evidence for its use in practical applications.

### B. HRV–stress model construction

In this study, the stress model was constructed using data from the Swell dataset, with a decision tree serving as the model algorithm. The training set from the Swell dataset was used for model training, and the validation set was utilized for performance evaluation. The trained decision tree model achieved an accuracy of 99.96%, indicating its effectiveness in predicting stress levels on the basis of changes in HRV parameters. To further assess the model's feasibility, precision, recall, and F1 scores were calculated for each stress level. For no stress, moderate stress, and severe stress, the precision values were 0.999 41, 0.999 64, and 0.999 58, respectively. The corresponding recall values were 0.999 58, 0.999 64, and 0.999 30, respectively. Similarly, F1 scores were high at 0.999 49, 0.999 64, and 0.999 44 for the three stress levels. These metrics demonstrate the excellent performance of the HRV–stress decision tree model, confirming its ability to accurately differentiate between stress levels and deliver reliable predictions. Overall, these results highlight the robustness of the decision tree model in stress prediction, indicating its potential for practical applications in stress monitoring systems.

### C. Evaluation of stress levels under different experimental conditions via a decision tree model

**FIG. 3.**
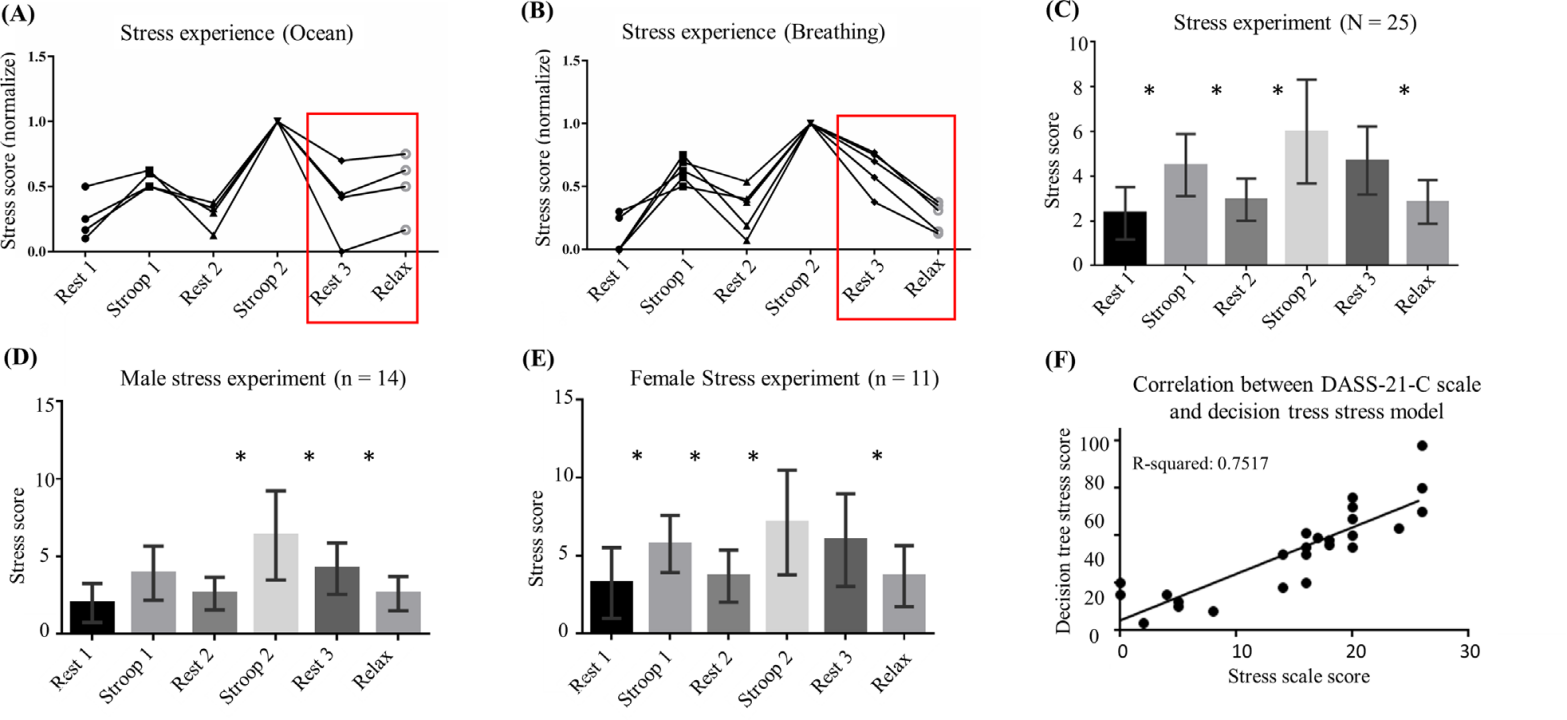

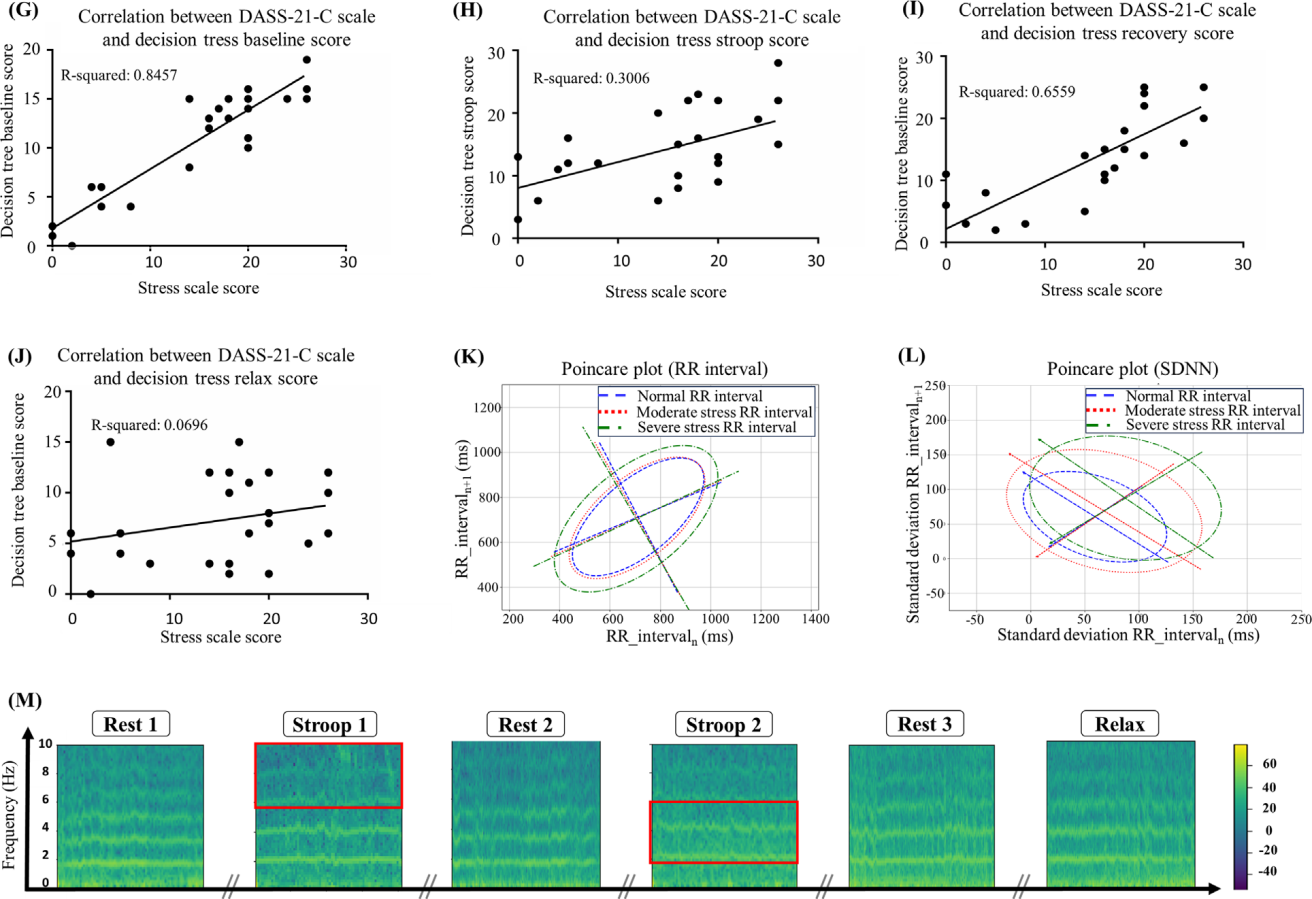
Decision tree performance evaluation. (a) Decision tree stress score trend (Stroop test with ocean video relaxation): The horizontal axis represents the experimental phases, and the vertical axis represents the normalized sum of the decision tree stress scores. During the Stroop 1 and Stroop 2 phases, the stress scores increase, indicating that the decision tree model effectively identifies the stress induced by the Stroop test. Interestingly, the stress scores also increase during the Relax phase, when the participants are watching a 10-minute ocean video for relaxation (see supplementary material Video 1). (b) Decision tree stress score trend (Stroop test with mindful breathing): This scenario is similar to that of (a), but mindful breathing is used for the relaxation phase instead of the ocean video (see supplementary material Video 2). The decision tree model shows a decreasing trend in stress, confirming the effectiveness of mindful breathing in stress reduction. As a result, mindful breathing is used for relaxation in future experiments. (c) Overall stress score trend: The horizontal axis represents the experimental phases, and the vertical axis represents the sum of the decision tree stress scores, showing the overall variation in stress across all phases. (d) Male stress score trend: This chart shows the stress score trend for male participants, with the horizontal axis representing the experimental phases and the vertical axis showing the sum of the stress scores. The graph highlights the variation in stress levels across different phases for male participants. (e) Female stress score trend: Similar to (d), this chart presents the stress score trend for female participants, showing variations in stress levels throughout the experimental phases. (f) Linear regression correlation between DASS-21-C scores and the decision tree model results: The horizontal axis represents DASS-21-C questionnaire stress scores, and the vertical axis represents the sum of the decision tree stress scores across all phases. The linear regression analysis reveals a strong positive correlation (correlation coefficient: 0.867, R-squared: 0.7517), indicating a strong relationship between the DASS-21-C scores and the model's stress scores. (g) Linear regression correlation between DASS-21-C scores and baseline scores. The horizontal axis represents DASS-21-C stress scores, and the vertical axis represents decision tree scores during the Rest 1 phase. A strong positive correlation is observed (correlation coefficient: 0.92, R-squared: 0.8457). (h) Linear regression correlation between DASS-21-C and Stroop scores. The horizontal axis represents DASS-21-C stress scores, and the vertical axis displays the sum of decision tree scores during the Stroop 1 and Stroop 2 phases. The correlation is moderate (correlation coefficient: 0.548, R-squared: 0.3006). (i) Linear regression correlation between DASS-21-C scores and recovery scores: The horizontal axis represents DASS-21-C stress scores, and the vertical axis represents the sum of decision tree scores during the Rest 2 and Rest 3 phases. A strong positive correlation is observed (correlation coefficient: 0.81, R-squared: 0.6559). (j) Linear regression correlation between the DASS-21-C score and relaxation score: The horizontal axis represents the DASS-21-C stress score, and the vertical axis represents the decision tree score during the Relax phase. A weak positive correlation is found (correlation coefficient: 0.264, R-squared: 0.0696). (k) Poincaré plot of RR intervals: This plot presents the Poincaré diagrams of RR intervals for 25 participants across different phases (Rest, Stroop 1, and Stroop 2). The horizontal and vertical axes represent the RR intervals between consecutive heartbeats, visually demonstrating how stress affects RR interval variability. The blue trajectories indicate no stress, the red trajectories indicate mild stress, and the green trajectories indicate severe stress. As the stress increases, the RR interval variability also increases. (l) Poincaré plot of the RR interval standard deviation: This plot shows the standard deviation of the RR intervals for the same 25 participants across different phases. The horizontal and vertical axes represent the standard deviations of consecutive RR intervals. As in (k), blue represents no stress, red represents mild stress, and green represents severe stress. With increasing stress, the RR interval variability becomes more pronounced, and greater deviations are observed. (m) PPG signal time–frequency analysis: This time–frequency analysis illustrates the changes in PPG signal characteristics across different experimental phases (Rest, Stroop, and Relax). The chart represents participants' physiological states and stress responses. During the Rest phase, the PPG signal is relatively stable, with a higher signal intensity in the range of 2–6 Hz and a lower intensity in the range of 6–10 Hz. During the Stroop 1 phase (highlighted in red), the 6–10 Hz signal intensity increases, whereas the 2–6 Hz signal becomes more unstable. As participants enter the Stroop 2 phase, the signal fluctuations become even more pronounced (as seen in the red Stroop 2 box). In the Rest 3 and Relax phases, the PPG signal stability gradually returns to levels similar to those in Rest 1.

In this study, a decision tree model was employed to evaluate the stress levels of participants under different experimental conditions. Two relaxation methods were tested: one using an ocean video and the other using mindfulness breathing. Figure 3(a) shows the stress variation trend during the ocean relaxation video, which included several phases, such as rest, the Stroop test, and relaxation. Stress levels significantly increased during both the Stroop 1 and Stroop 2 phases, confirming the effectiveness of the Stroop test in inducing stress. However, during the ocean relaxation video, stress levels continued to rise, indicating that this video was ineffective in reducing stress. In contrast, Fig. 3(b) illustrates the results of mindfulness breathing, where stress levels decreased during the relaxation phase, confirming the effectiveness of this method. Consequently, mindfulness breathing was chosen for subsequent experiments.

Throughout the stress experiment, participants experienced noticeable changes in stress levels across the different phases, demonstrating the decision tree model's effectiveness in stress assessment. Figure 3(c) provides a statistical analysis of the stress levels across the experimental phases. Stress levels were lower during the resting phase than during the Stroop phase, and significant differences were observed among most groups (*p* < 0.05). Notably, there was no significant difference between Stroop 2 and Rest 3, likely due to the need for a longer recovery time from high stress. However, guided relaxation significantly reduced the stress level (*p* < 0.05), confirming the effectiveness of the relaxation method. These results indicate that the decision tree model effectively differentiates stress levels across various conditions. Figures 3(d) and 3(e) explore sex differences in stress trends. Significant differences in males were observed between Rest 2 and Stroop 2, Stroop 2 and Rest 3, and Rest 3 and Relax (*p* < 0.05), whereas females showed no significant difference between Stroop 2 and Rest 3. The HRV parameter analysis revealed that, compared with females, males exhibited better self-recovery under high stress. Significant changes in HRV parameters such as the mean RR, SDNN, RMSSD, LF, HF, and LF/HF were observed in males, whereas females presented significant changes under mild stress and weaker recovery after high stress, particularly in terms of RMSSD, HF, and LF/HF.

Figure 3(f) shows the linear regression analysis of the DASS-21-C scale and the decision tree stress model. The overall stress scores were strongly positively correlated with the DASS-21-C scale scores (r = 0.867, R^2^ = 0.7517). Figure 3(g) shows the correlation between the DASS-21-C scale score and the baseline score (Rest 1), which was even stronger (r = 0.92, R^2^ = 0.8457). Figure 3(h) shows the correlation during the Stroop phases, indicating moderate correlation (r = 0.548, R^2^ = 0.3006). Figure 3(1) shows the recovery scores, which are strongly correlated (r = 0.810, R^2^ = 0.6559), whereas Fig. 3(j) shows weak correlation during the Relax phase (r = 0.264, R^2^ = 0.0696). Figures 3(k) and 3(l) present Poincaré plots of the RR intervals and their standard deviations under different stress levels. The blue-, red-, and green-dashed lines represent the RR intervals for no stress, moderate stress, and severe stress, respectively. The Poincaré plot is an HRV analysis tool that is commonly used to visualize the nonlinear dynamics of time series. The elliptical contours become more dispersed under moderate and severe stress, indicating increased HRV variability with increasing stress levels. These findings support the conclusion that HRV parameters can be used to quantify stress effectively.

Finally, Fig. 3(m) presents the time–frequency signals for each phase. The time–frequency plots of the PPG signals show stable characteristics during Rest 1, Rest 2, and Rest 3, with dominant frequencies in the 2–6 Hz range. During Stroop 1 and Stroop 2, the signal strength in the 6–10 Hz range increases, especially during Stroop 2, reflecting heightened stress. During the Relax phase, the time–frequency signals return to a stable state similar to those in the rest phases, confirming that relaxation reduced stress and restored normal signal patterns.

### D. Effects of the stress response on the HRV parameters

**FIG. 4.**
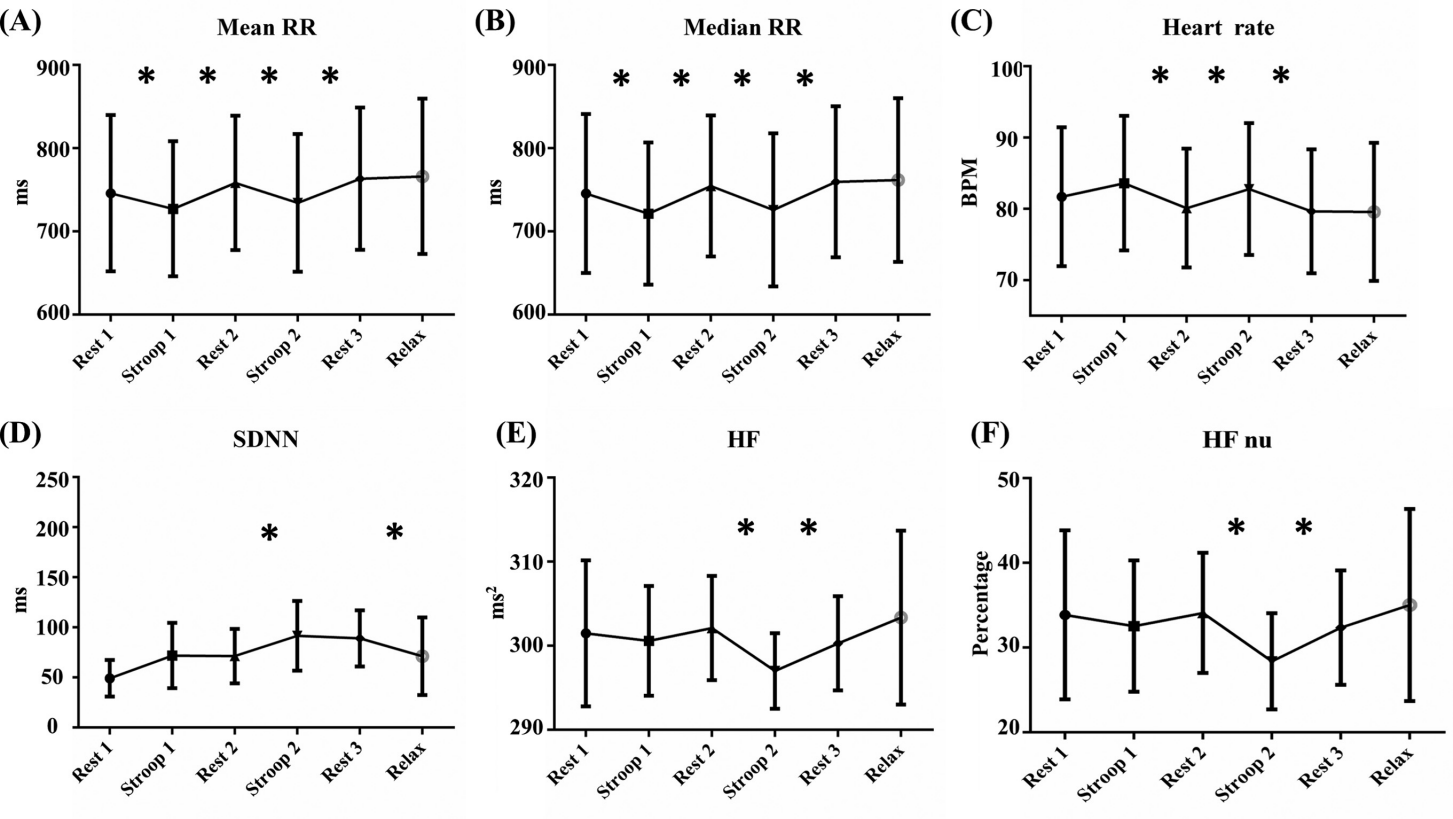

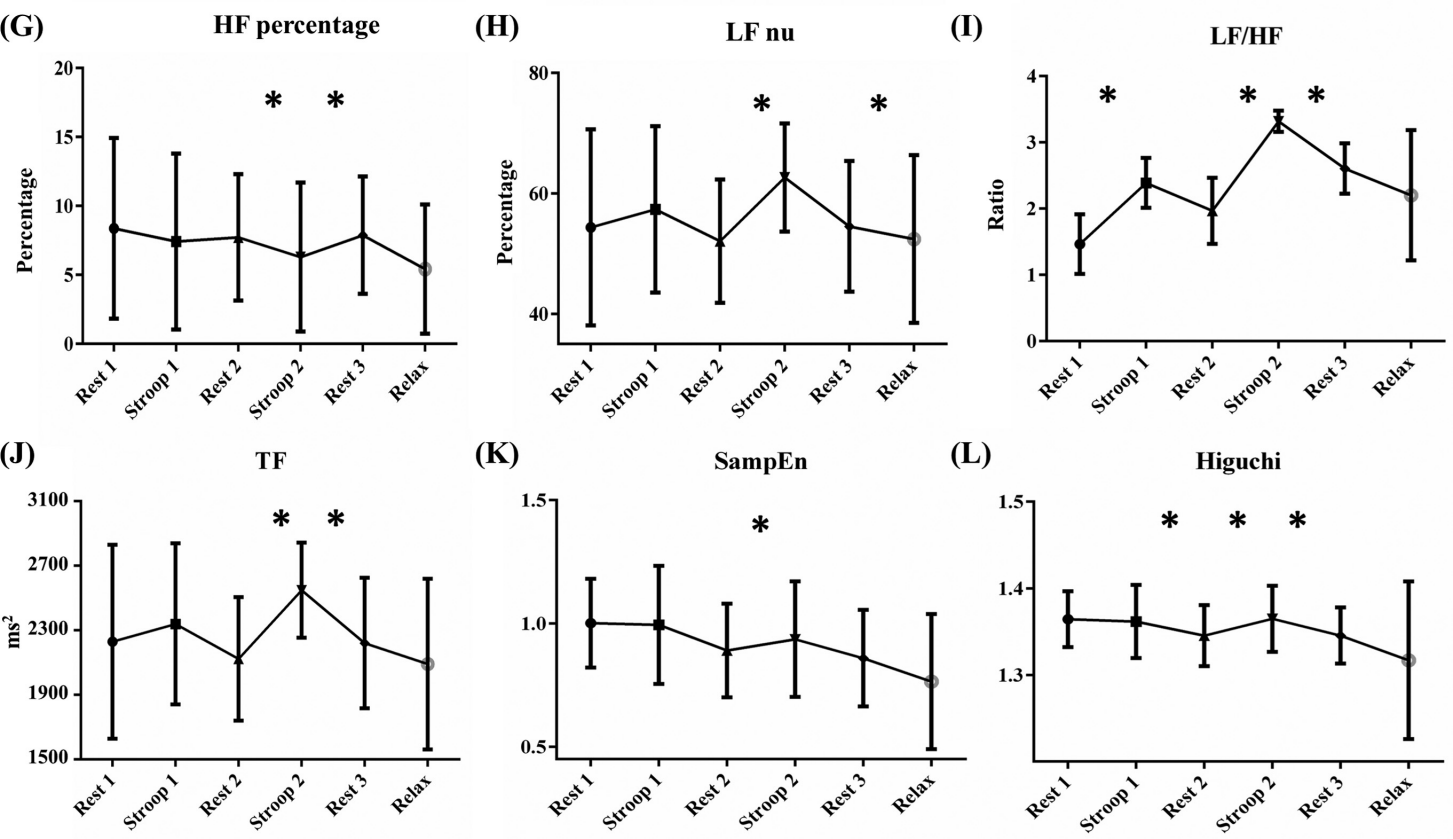
Trend changes in HRV parameters with significant differences. (a) Mean RR trend: The mean RR is the average of all RR intervals calculated over 30 s, reflecting overall heart activity and rhythm stability. Shorter mean RR intervals indicate increased sympathetic activity, whereas longer intervals suggest increased parasympathetic activity. During the Stroop phases, the mean RR is significantly lower than that during the Rest phases, indicating that sympathetic activity is elevated in response to external stress. (b) Median RR trend: The median RR represents the median value of all RR intervals calculated over 30 s and reflects the body's ability to adapt to stress. A shorter median RR during stress suggests heightened sympathetic activity. The median RR is lower during the Stroop phases than in the Rest phases, indicating significant differences and further confirming the increase in sympathetic activity under stress. (c) Heart rate trend: The heart rate, calculated from RR intervals over 30 s, is given in terms of the number of beats per minute (bpm). It reflects the physiological response to stress, as the sympathetic nervous system increases the heart rate under stress or perceived threat. The heart rate significantly increases during the Stroop phases, with notable differences between Stroop 1 and Rest 2, Rest 2 and Stroop 2, and Stroop 2 and Rest 3. (d) SDNN trend: SDNN is the standard deviation of all RR intervals over 30 s and reflects overall heart rate variability. Higher SDNN values indicate more stable heart rhythms, whereas lower values indicate increased instability. SDNN decreases during the Stroop phase compared with that in the Rest phases but significantly increases during the Relax phase, indicating recovery and relaxation. (e) HF trend: High-frequency (HF) values reflect heart rate variability in the high-frequency range and is associated with parasympathetic nervous system activity. HF decreases during the Stroop phases compared with that in the Rest phases, indicating parasympathetic suppression under psychological stress, with particularly significant differences around Stroop 2. (f) HF nu trend: HF nu represents the proportion of HF remaining after subtracting VLF from TF and is used to assess parasympathetic activity under different conditions. HF nu decreases during the Stroop phases, confirming the suppression of parasympathetic activity under stress, with significant differences around Stroop 2. (g) HF percentage trend: HF percentage represents the proportion of heart rate variability in the high-frequency range relative to the total heart rate variability. Changes in HF percentage during the stress experiment indicate the relative contribution of parasympathetic activity to overall heart rate variability. HF percentage shows a downward trend during the Stroop phases, with significant differences around Stroop 2. (h) LF nu trend: LF nu represents the proportion of the low-frequency (LF) value that remains after subtracting VLF from TF and is associated with sympathetic nervous system activity. Significant differences in LF nu are observed between Rest 2 and Stroop 2 and between Rest 3 and the Relax phase, indicating that sympathetic activity changes in response to varying stress levels. (i) LF/HF ratio trend: The LF/HF ratio reflects the balance between sympathetic and parasympathetic nervous system activity. This ratio increases during the Stroop phases compared with that in the Rest phases, indicating a stress-induced shift in autonomic balance, with significant differences between Rest 1 and Stroop 1 and around Stroop 2. (j) TF trend: The total frequency (TF) represents total heart rate variability across all frequency ranges. TF increases significantly during the Stroop phases compared with that in the Rest phases, highlighting the effect of psychological stress on overall heart rate variability, with particularly significant differences around Stroop 2. (k) SampEn trend: Sample entropy (SampEn) measures the complexity and unpredictability of heart rate signals. SampEn decreases during the Rest and Relax phases, possibly indicating that relaxation promotes more regular heart rhythms. Significant differences are noted between Rest 2 and Stroop 2, where signal complexity increases under severe stress. (l) Higuchi trend: The Higuchi algorithm calculates the fractal dimension of heart rate signals, reflecting their complexity. The results show that the Higuchi dimension increases during the Stroop phases compared with that in the Rest phases, suggesting greater signal complexity under stress. Significant differences are observed between Stroop 1 and Rest 2 and between Rest 2 and Stroop 2. (* indicates significant differences, *p* < 0.05.)

Figure 4 shows the trends of the HRV parameters with significant differences (*p* < 0.05) across the various experimental phases. The detailed HRV parameter variations are presented in Table I, including the mean RR [Fig. 4(a)], median RR [Fig. 4(b)], heart rate [Fig. 4(c)], SDNN [Fig. 4(d)], HF [Fig. 4(e)], HF nu [Fig. 4(f)], HF percentage [Fig. 4(g)], LF nu [Fig. 4(h)], LF/HF [Fig. 4(1)], TF [Fig. 4(j)], SampEn [Fig. 4(k)], and Higuchi [Fig. 4(l)]. During the Stroop phases (Stroop 1 and Stroop 2), the mean RR and median RR significantly decreased (*p* < 0.05), indicating increased heart rate and stress levels. These parameters returned to near-resting levels during the Relax phase, indicating the positive impact of relaxation on RR intervals. Heart rate followed a similar trend, showing significant differences between Stroop 1, Rest 2, Stroop 2, and Rest 3 (*p* < 0.05) as well as stress-induced increases in heart rate. SDNN, which reflects overall heart rate variability, significantly differed between Rest 2 and Stroop 2 and between Rest 3 and the Relax phase (*p* < 0.05). SDNN experienced the greatest changes during intense stress and relaxation phases, indicating its sensitivity to both stress and recovery.

**TABLE I.** List of HRV parameters used in the stress experiment. In this experiment, a total of 34 HRV parameters were analyzed. PPG signals were recorded during each experimental phase, and the data were analyzed every 30 s to extract HRV parameters. These parameters include 15 time-domain parameters, 8 relative values, and 11 frequency-domain parameters. A statistical analysis was performed by comparing adjacent experimental phases (e.g., Rest 1 vs Stroop 1, Stroop 1 vs Rest 2) to observe changes in HRV parameters under different stress levels. The parameters that showed significant differences included the mean RR, median RR, SDNN, HR, LF nu, HF, HF percentage, HF nu, total power, LF/HF, SampEn, and Higuchi. By analyzing the changes in these parameters, this study further explored the interaction between HRV and stress, shedding light on the body's adaptation mechanisms and physiological responses to stress. (* indicates a significant difference, *p* < 0.05.)

	Rest 1	Stroop 1	Rest 2	Stroop 2	Rest 3	Relax
Mean RR	745.93 ± 93.84	**727.16 ± 81.11** ^*^	**758.49 ± 80.75** ^*^	**734.35 ± 82.70** ^*^	**763.31 ± 85.48** ^*^	766.25 ± 93.48
Median RR	745.66 ± 95.47	**721.49 ± 85.34** ^*^	**754.61 ± 84.68** ^*^	**725.84 ± 91.98** ^*^	**759.66 ± 90.67** ^*^	761.77 ± 98.45
SDNN	59.54 ± 26.23	66.62 ± 36.24	71.57 ± 26.90	84.88 ± 36.89	79.99 ± 29.42	**94.73 ± 45.04** ^*^
RMSSD	79.77 ± 42.50	93.86 ± 55.57	97.03 ± 46.09	124.24 ± 58.48	110.40 ± 49.47	133.65 ± 76.76
SDSD	79.65 ± 42.44	93.79 ± 55.48	96.91 ± 46.03	124.13 ± 58.40	110.28 ± 49.44	133.44 ± 76.65
SDNN_RMSSD	59.54 ± 26.23	66.62 ± 36.24	71.57 ± 26.90	84.88 ± 36.89	79.99 ± 29.42	94.73 ± 45.04
HR	81.70 ± 9.74	83.61 ± 9.43	**80.10 ± 8.33** ^*^	**82.78 ± 9.25** ^*^	**79.65 ± 8.68** ^*^	79.56 ± 9.69
pNN25	63.84 ± 17.03	65.89 ± 18.98	68.17 ± 14.88	71.82 ± 13.39	70.31 ± 15.21	72.72 ± 14.24
pNN50	37.53 ± 18.30	39.97 ± 24.29	42.86 ± 18.53	48.83 ± 20.23	46.58 ± 20.23	52.07 ± 20.96
SD1	56.32 ± 30.01	66.32 ± 39.23	68.52 ± 32.55	87.77 ± 41.30	77.98 ± 34.96	94.36 ± 54.20
SD2	59.74 ± 22.51	65.92 ± 33.77	72.37 ± 22.48	79.16 ± 32.16	78.98 ± 25.62	92.24 ± 39.57
Kurt	1.11 ± 1.35	1.93 ± 3.68	1.49 ± 2.08	2.90 ± 4.86	1.48 ± 2.19	1.18 ± 3.62
Skew	0.18 ± 0.39	0.50 ± 0.80	0.34 ± 0.55	0.77 ± 1.22	0.40 ± 0.57	0.42 ± 0.82
Rel mean RR	0.00 ± 0.00	0.00 ± 0.00	0.00 ± 0.00	0.00 ± 0.00	0.00 ± 0.00	0.00 ± 0.01
Rel median RR	0.01 ± 0.01	0.00 ± 0.01	0.01 ± 0.01	0.01 ± 0.01	0.01 ± 0.01	0.01 ± 0.02
Rel SDNN	0.10 ± 0.05	0.12 ± 0.07	0.12 ± 0.06	0.16 ± 0.07	0.14 ± 0.07	0.17 ± 0.10
Rel RMSSD	0.11 ± 0.05	0.12 ± 0.07	0.12 ± 0.06	0.16 ± 0.07	0.14 ± 0.07	0.17 ± 0.10
Rel SDSD	0.18 ± 0.10	0.21 ± 0.13	0.21 ± 0.11	0.28 ± 0.13	0.24 ± 0.12	0.29 ± 0.18
Rel SDNN_RMSSD	0.10 ± 0.05	0.12 ± 0.07	0.12 ± 0.06	0.16 ± 0.07	0.14 ± 0.07	0.17 ± 0.10
Rel Kurt	0.69 ± 0.90	1.05 ± 1.96	1.11 ± 1.29	1.72 ± 2.66	1.05 ± 1.51	0.85 ± 2.15
Rel Skew	−0.23 ± 0.21	−0.11 ± 0.19	−0.12 ± 0.19	−0.21 ± 0.30	−0.25 ± 0.17	−0.25 ± 0.17
VLF	878.89 ± 920.79	1064.49 ± 1166.45	979.33 ± 1023.90	1644.64 ± 1324.04	1421.56 ± 1334.07	1144.23 ± 12,45.59
VLF percentage	52.46 ± 25.46	50.76 ± 15.44	51.34 ± 21.59	58.05 ± 21.79	57.05 ± 47.69	52.68 ± 30.17
LF	472.98 ± 523.29	726.63 ± 610.00	614.22 ± 733.11	912.69 ± 1120.06	772.99 ± 862.86	706.63 ± 1086.10
LF percentage	28.23 ± 32.34	34.66 ± 37.34	32.20 ± 34.08	32.21 ± 37.77	31.02 ± 35.19	32.53 ± 26.03
LF nu	59.38 ± 6.55	70.39 ± 6.38	66.16 ± 4.58	**76.78 ± 5.41** ^*^	72.22 ± 4.26	**68.74 ± 4.69** ^*^
HF	323.48 ± 89.68	305.59 ± 68.53	314.11 ± 65.20	**276.00 ± 45.48** ^*^	**297.29 ± 56.59** ^*^	321.35 ± 103.33
HF percentage	19.30 ± 16.28	14.57 ± 13.81	16.47 ± 10.25	**9.74 ± 8.98** ^*^	**11.93 ± 10.87** ^*^	14.79 ± 13.93
HF nu	40.61 ± 9.97	29.61 ± 7.75	33.84 ± 7.09	**23.22 ± 5.68** ^*^	**27.78 ± 6.74** ^*^	31.26 ± 11.34
Total power	1675.35 ± 1252.01	2096.71 ± 1584.48	1907.66 ± 1691.57	**2833.33 ± 1764.36** ^*^	**2491.84 ± 1680.12** ^*^	2172.21 ± 1397.21
LF/HF	1.46 ± 0.44	**2.38 ± 0.37** ^*^	1.96 ± 0.48	**3.31 ± 0.16** ^*^	**2.60 ± 0.37** ^*^	2.20 ± 0.96
HF/LF	0.68 ± 0.17	0.42 ± 0.14	0.51 ± 0.21	0.30 ± 0.18	0.38 ± 0.21	0.45 ± 0.31
SampEn	1.00 ± 0.18	0.99 ± 0.24	**0.89 ± 0.19** ^*^	0.94 ± 0.23	0.86 ± 0.20	0.76 ± 0.27
Higuchi	1.36 ± 0.03	1.D36 ± 0.04	**1.35 ± 0.04** ^*^	**1.36 ± 0.04** ^*^	**1.35 ± 0.03** ^*^	1.32 ± 0.09

The parasympathetic-related parameters, HF and HF nu, significantly decreased during the Stroop phase (*p* < 0.05), suggesting reduced parasympathetic activity under stress. These parameters increased during the Relax phase (p < 0.05), reflecting effective recovery through relaxation. The HF percentage followed a similar trend, decreasing under stress and increasing during relaxation (*p* < 0.05). The sympathetic-related parameters, LF nu and LF/HF, increased significantly during the Stroop phase (*p* < 0.05), indicating heightened sympathetic activity in response to stress. Both parameters decreased during the Relax phase (*p* < 0.05), demonstrating a return to balance with reduced sympathetic influence. Total power (TF) also significantly increased during the Stroop phase (*p* < 0.05), reflecting overall stimulation of the autonomic nervous system, particularly sympathetic activation. TF significantly decreased during the Relax phase (*p* < 0.05), indicating a shift toward parasympathetic dominance. SampEn and Higuchi, both measures of heart rate signal complexity, showed increased values during the Stroop phase (*p* < 0.05), indicating greater heart rate signal complexity under stress.^41^ Conversely, during the Relax phase, SampEn and Higuchi significantly decreased (*p* < 0.05), indicating a return to more regular heart rate dynamics post-relaxation.

## III. DISCUSSION

In this study, a wearable device was developed based on PPG technology for noninvasive measurement of physiological signals and analysis of HRV parameters. These parameters, including time-domain, frequency-domain, and nonlinear parameters, were used to quantify individual stress levels. Previous studies have also employed empirical mode decomposition (EMD) for HRV analysis,^42^ and this method may be integrated into the system developed in this study in the future to further improve the model's ability to quantify stress. The results demonstrate that PPG signals can effectively replace traditional ECG signals for HRV parameter calculation, with HRV measures showing variations across different stress conditions. The HRV–stress model was able to determine the subject's stress state on the basis of HRV changes, confirming the model's accuracy and reliability. Initially, the MAX86150 sensor was used to verify the feasibility of using PPG signals to compute HRV parameters. As shown in Fig. 1(d), there was a strong positive correlation between the RR intervals derived from the PPG and ECG signals (R-squared = 0.9837), confirming that PPG signals can serve as a reliable substitute for ECG signals in HRV calculations. To further validate the accuracy of the PPG signals collected by the self-developed wearable device, RR interval, time-domain, and frequency-domain analyses were performed. The results, illustrated in Fig. 2(d), show a high positive correlation (R-squared = 0.8401) between the RR intervals obtained from the self-developed device and from the MAX86150 sensor. Time-domain and frequency-domain analyses, as depicted in Figs. 2(e) and 2(f), respectively, also revealed strong similarities between the two devices, with high positive correlations in both the time-domain (correlation = 0.8965) and frequency-domain (correlation = 0.7817) analyses. These findings support the feasibility of using PPG signals from the developed wearable device for HRV parameter calculation. To construct the HRV–stress model, the Swell dataset was utilized for training and validation. The choice of a decision tree algorithm was made after careful evaluation of both performance requirements and practical constraints. While various machine learning architectures have been explored with the SWELL dataset, the real-time nature of stress quantification requires balancing accuracy with computational efficiency. Multiple research approaches have been tested with the SWELL dataset. Androutsou *et al.* achieved 90.01% accuracy with random forests,^43^ while Ghosh *et al.* used CNNs to achieve 94.8% accuracy.^44^ However, these models require significant computational resources and processing time. In contrast, our decision tree implementation achieved an accuracy of 99.96%, while maintaining faster execution times and lower resource requirements.^45^ The model's effectiveness was further confirmed through additional evaluation metrics, including precision, recall, and F1 score. The decision tree's simpler structure allows for rapid execution and lower hardware requirements, making it particularly well-suited for continuous stress monitoring. Additionally, its interpretable nature provides clear insights into the decision-making process, a valuable feature for both development and clinical applications.^46^

While various wearable systems have been developed for stress monitoring studies, a key limitation in many existing approaches is the lack of psychological validation of stress measurements. This study addresses this critical gap by incorporating clinical psychological assessment through the DASS-21-C questionnaire. Recent studies in stress monitoring illustrate this limitation. For instance, McDuff *et al.* developed a wearable PPG-based stress detection system achieving 86.0% accuracy but relied solely on physiological parameters without psychological validation.^47^ Similarly, Anusha *et al.* presented a multi-sensor stress monitoring device that showed promising results in detecting stress-induced physiological changes achieving 95.86% accuracy yet lacked correlation with standardized psychological stress measures.^48^ Cho *et al.* introduced an innovative approach combining PPG and thermal imaging for stress detection. While their system achieved 78.3% accuracy and included momentary self-reported stress levels,^49^ it still lacked validation against comprehensive clinical psychological assessments. This highlights a common limitation in the field where even advanced multi-modal approaches often rely on simplified self-report measures rather than validated clinical instruments. The integration of psychological and physiological measurements distinguishes this approach from existing methods. While Iqbal *et al.* utilized machine learning for stress detection with 92.4% accuracy using PPG and EDA signals, their validation focused exclusively on physiological responses to controlled stressors.^50^ In contrast, the system demonstrates strong correlation with DASS-21-C scores (R^2^ = 0.92 for baseline measurements), providing crucial psychological context to physiological stress indicators. This study's strong correlation between HRV parameters and DASS-21-C scores establishes a more robust framework for stress quantification that considers both physiological and psychological aspects of the stress response.

**FIG. 5.**
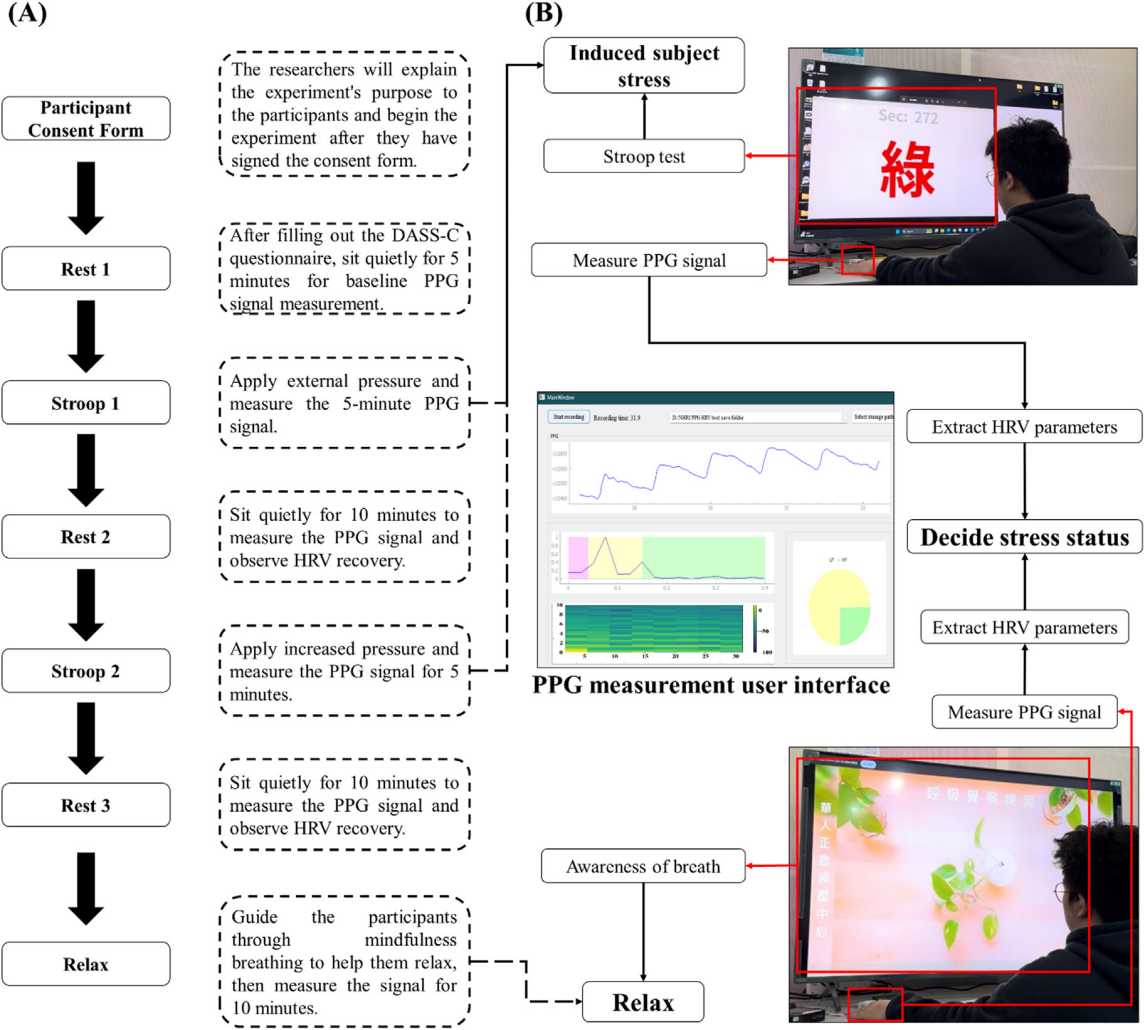
HRV–stress model validation experiment flow chart. (a) Experimental flow chart: Before the experiment begins, the participants are briefed on the experimental process and asked to sign a consent form. The experiment is divided into six phases: Rest 1, Stroop 1, Rest 2, Stroop 2, Rest 3, and Relax. During the Rest 1 phase, participants first complete the DASS-21-C questionnaire and then sit quietly for five minutes while their PPG signals are measured to establish a baseline. Next, the Stroop test is administered as a stress-inducing stimulus. The Stroop test, a well-established psychological method for eliciting short-term stress, is conducted for five minutes while PPG signals are recorded; this phase is classified as mild stress. Afterward, the participants rest quietly for ten minutes while continued PPG measurements are taken to observe HRV recovery under mild stress. The participants then undergo another five-minute Stroop test, this time with added auditory interference to simulate more severe stress. PPG signals are measured during this phase, which is defined as severe stress due to the additional stimuli. The participants then rest quietly for another ten minutes while their PPG signals are measured to observe HRV recovery under severe stress. Finally, the participants watch a ten-minute relaxation video while PPG signals are recorded to determine whether the HRV parameters significantly change in response to external relaxation stimuli. (b) Actual experimental setup: In the experimental setup, a screen positioned in front of the participant delivers stress or relaxation stimuli. The participant wears a finger-clip device on their left index finger to measure the PPG signals. The HRV parameters are calculated from the PPG signals every 30 s, and both the raw PPG data and HRV parameters are automatically saved in separate CSV files. These HRV parameters can later be input into the decision tree model to determine stress variation trends during the experiment. Multimedia available online.10.1063/5.0256590.1

After the hardware and model were validated, system integration was conducted. The PPG signals were measured, and the HRV parameters were calculated simultaneously through a self-developed graphical user interface (GUI). This GUI enables real-time acquisition of PPG signals and calculates HRV parameters every 30 s. Once the calculations are complete, the HRV parameters are automatically input into the HRV–stress model for analysis, and the results are instantly displayed on the GUI. To validate the system's effectiveness and practicality, a series of experiments were designed to test the model's performance under various stress stimuli. The experimental procedure is illustrated in Fig. 5 (Multimedia view); participants used the wearable device to measure PPG signals in baseline, stressful, and relaxed states. The experimental results are presented in Fig. 3. Figures 3(a) and 3(b) show the trends of the results and compare the effectiveness of different relaxation videos in alleviating stress. The trends clearly indicate that stress levels during the Stroop phase were greater than those during the Rest phase and that stress levels during Stroop 2 were greater than those during Stroop 1, suggesting that the Stroop test with added sound interference induced a more intense stress state. In the final relaxation phase, the participants watched ocean scenes and practiced mindful breathing. Figure 3(a) shows an upward trend in stress levels from Rest 3 to Relax, whereas Fig. 3(b) displays a downward trend. These findings indicate that mindful breathing effectively reduces stress, a conclusion supported by previous literature.^51–53^ Figure 3(c) presents the complete results of the stress experiment, which reveal no significant differences in stress levels between the Stroop 2 and Rest phases. This may be attributed to individuals' low self-recovery efficiency under high stress, which prevents them from relaxing quickly within a short time frame.^54^ Figures 3(d) and 3(e) show the stress variation trends for males and females. According to the experimental results, males exhibited a better recovery ability when responding to acute stress.^55,56^ As shown in Fig. 3(d), significant differences in stress levels for males were observed between the Rest 2 and Stroop 2, Stroop 2 and Rest 3, and Rest 3 and Relax phases. In contrast, Fig. 3(e) shows that for females, significant stress level differences were found in phases other than between Stroop 2 and Rest 3. These results suggest that males do not exhibit significant stress changes under mild stress (Stroop 1) and recover more effectively from severe stress (Stroop 2). On the other hand, females experienced significant stress changes under mild stress (Stroop 1) but showed no significant differences between Stroop 2 and Rest 3, indicating that females have poorer self-regulation under severe stress and may require external stimuli for effective relaxation. There were also notable sex differences in HRV parameter changes. Males exhibited a more significant recovery trend in terms of both time-domain and frequency-domain HRV parameters, such as the mean RR, SDNN, RMSSD, LF, HF, and LF/HF, when responding to acute stress. These findings indicate that males can restore heart rhythm more quickly after severe stress, indicating stronger regulation between the sympathetic and parasympathetic nervous systems. On the other hand, females presented significant changes in HRV parameters under mild stress (Stroop 1), including time-domain measures such as HR, mean RR, SDNN, and RMSSD, as well as frequency-domain parameters such as LF, HF, and LF/HF. These findings suggest that mild stress significantly affects the autonomic nervous system in females. However, their HRV recovery after severe stress (Stroop 2) was not as pronounced as that in males, with the RMSSD, HF, and LF/HF parameters indicating weaker self-recovery abilities. These findings suggest that females have more difficulty quickly returning to a stable state after experiencing severe stress. Males are able to recover more quickly following acute and intense stress, likely due to rapid regulation of the sympathetic nervous system and stress hormones such as cortisol. In contrast, females' stress responses are influenced by endocrine factors, such as estrogen, which may contribute to longer recovery times. These physiological differences help explain the sex differences in coping with acute stress.^56^ Examining the physiological and hormonal mechanisms underlying these sex-based differences in HRV responses reveals complex interactions between the autonomic nervous system and endocrine factors.^57^ In males, the more efficient stress recovery appears to be mediated by rapid catecholamine clearance and more effective hypothalamic-pituitary-adrenal (HPA) axis regulation.^58^ The quicker restoration of sympathetic-parasympathetic balance observed in male participants' HRV parameters (particularly in SDNN and LF/HF ratios) suggests more efficient autonomic nervous system adaptation after acute stress exposure. This may be attributed to the influence of androgens on cardiac autonomic control and stress hormone metabolism.^59,60^ In contrast, the female stress response pattern, characterized by heightened sensitivity to mild stress and prolonged recovery periods, likely reflects the modulatory effects of estrogen and progesterone on autonomic function.^61^ These sex hormones influence both baseline autonomic tone and stress-induced autonomic responses through multiple mechanisms,^62^ including direct effects on cardiac receptors and indirect modulation of neurotransmitter systems. The slower recovery in HRV parameters (especially RMSSD and HF components) observed in female participants may be partially explained by estrogen's effects on vagal tone and its interaction with stress hormone signaling pathways.^63,64^ Additionally, research has shown that estrogen can enhance stress sensitivity by modifying neurotransmitter receptor expression and function in autonomic control centers. Understanding these sex-specific physiological mechanisms is crucial for developing targeted interventions and personalized stress management strategies. The observed differences in HRV recovery patterns suggest that stress management approaches may need to be tailored differently for males and females,^65^ particularly in clinical settings where stress monitoring plays a crucial role in treatment.^66^

One of the key innovations of this study is that, in addition to quantifying stress through engineering methods, it also quantifies stress via clinical psychology questionnaires. Figures 3(f)–3(j) show the results of linear regression analyses between the questionnaire-based stress scores and model-based stress scores. Since there are currently no established methods for quantifying stress comparisons, this study performs linear regression correlation analysis between the stress scores from the DASS-21-C questionnaire and the following variables: total stress values across all phases, baseline stress value (Rest 1), total stress values during the Stroop phases (Stroop 1, Stroop 2), total stress values during the recovery phases (Rest 2, Rest 3), and stress values in the relaxation phase (Relax). The varying correlations observed across different experimental phases warrant detailed discussion, particularly considering our study's methodological context and limitations. Traditional approaches to stress assessment using individual HRV parameters have typically shown relatively weak correlations with psychological questionnaires. For example, previous studies examining direct correlations between single HRV parameters and psychological stress scales have reported correlation coefficients ranging from 0.01 to 0.2, indicating only low relationships.^67^ Our machine learning approach, which integrates multiple HRV parameters into a unified model, demonstrates significantly improved correlation with psychological assessments. While our relatively small sample size (n = 25) and homogeneous participant characteristics may influence the stability of correlation estimates, several meaningful patterns emerge from our analysis. The strongest correlation was found during baseline measurements (correlation coefficient = 0.92, R^2^ = 0.8457), reflecting a robust relationship between physiological and psychological stress indicators under natural conditions. This marked improvement over traditional single-parameter approaches suggests that machine learning can effectively capture the complex, multivariate nature of stress responses that may not be apparent when examining individual HRV parameters in isolation. The baseline phase, free from external stressors, provides a more accurate representation of an individual's intrinsic stress state, aligning closely with the DASS-21-C's assessment of stress levels over the previous week. Similarly, the recovery phase stress value (correlation coefficient = 0.81) showed strong positive correlation with the DASS-21-C score, suggesting that recovery periods may better reflect underlying stress states. These stronger correlations during stable phases might be partially attributed to our relatively homogeneous sample of healthy young adults (mean age 24.8 ± 2.9 y), as individual variations in baseline stress responses may be more constrained within this demographic. Notably, the Stroop phases showed a moderate correlation (correlation coefficient = 0.548, R^2^ = 0.3006), while the relaxation phase exhibited the weakest correlation (correlation coefficient = 0.264, R^2^ = 0.0696). These lower correlations during active intervention phases are particularly informative and may reflect both the inherent variability in individual stress responses and the limited sample size's impact on capturing the full range of possible stress reactions. The moderate correlation during the Stroop test suggests that acute cognitive stress induces physiological responses that may temporarily deviate from an individual's baseline stress state as measured by DASS-21-C. The even lower correlation during the relaxation phase (R^2^ = 0.0696) indicates that guided relaxation techniques can effectively modify physiological stress responses regardless of an individual's underlying stress level. This finding has important implications for stress management interventions, suggesting that relaxation techniques may be universally beneficial, independent of baseline stress conditions. A final linear regression analysis combining all the stress scores revealed a strong positive correlation (correlation coefficient = 0.867), supporting the overall validity of the model. The significance of these findings lies in the fact that the developed stress model is connected with traditional psychological questionnaires, which gives the model psychological relevance. The pattern of varying correlations—strongest during baseline, moderate during stress induction, and lowest during relaxation—suggests that our system effectively captures both stable stress states and dynamic stress responses to external interventions, despite the potential limitations of our sample size. This dual capability makes the system particularly valuable for both diagnostic assessment and therapeutic monitoring. Future studies with larger and more diverse populations would be valuable to validate these preliminary findings and establish more generalizable relationships between acute stress responses and psychological stress measures. Subsequent analyses examined the impact of different stress levels on the HRV parameters. In Figs. 3(1) and 3(j), Poincaré plots are used to analyze the effects of psychological stress on RR interval variability by comparing RR intervals and their standard deviations. The Poincaré plot, a nonlinear dynamic analysis tool, shows the distribution patterns and density of scattered points, clearly illustrating the changes in RR intervals under varying stress levels. As stress increases, RR interval variability increases significantly, and the scatter points become more dispersed, indicating increased irregularity in heart rhythm. Figure 3(k) shows the time–frequency variations in the PPG signals during the experiment. During different stress phases, the spectrograms reveal changes in frequency components. Under stress-free conditions, the spectrogram indicates more stable low-frequency components, whereas under stress, the energy of the high-frequency components increases significantly, reflecting the activation of the sympathetic nervous system and the physiological effects of stress. In this study, a total of 34 HRV parameters were analyzed, as summarized in Table I. Among these, 12 HRV parameters exhibited significant differences across different stress phases (*p* < 0.05). The parameters showing significant differences are displayed in the charts in Fig. 4. Examining the transitions from Rest 1 to Stroop 1 and from Rest 2 to Stroop 2, it is found that nine parameters exhibited significant changes only during the transition from Rest 2 to Stroop 2. These parameters include SDNN [Fig. 4(d)], HF [Fig. 4(e)], HF percentage [Fig. 4(g)], LF nu [Fig. 4(h)], LF/HF [Fig. 4(1)], TF [Fig. 4(j)], SampEn [Fig. 4(k)], and Higuchi [Fig. 4(l)]. Among these, one is a time-domain parameter, five are frequency-domain parameters, and two are nonlinear parameters. The changes in these parameters provide insights into the stronger autonomic nervous system response during intense stress, as reflected in the frequency-domain and nonlinear parameters.^68^

This study establishes a method for quantifying psychological stress, with the stress scores calculated by the model during the baseline phase showing a strong positive correlation with traditional psychological stress scales. These findings indicate that the model developed in this study is reasonably effective in assessing psychological stress. However, this finding warrants further discussion regarding several key limitations and future research directions. While the current sample size of 25 participants (mean age 24.8 ± 2.9 y) allowed for detailed individual monitoring and thorough validation of the system's capabilities, it represents a relatively homogeneous group that may not fully capture the physiological and psychological stress responses across different demographic populations. The study's limitations extend to both participant characteristics and experimental design. The stress induction method—the Stroop test—was applied in a laboratory environment and represents only a single stressor, which may not fully reflect the complexity of real-world stress situations. Furthermore, all participants were healthy individuals without mental health conditions, limiting the current understanding of how the system might perform in clinical populations. To address these limitations, future research should pursue multi-center validation studies incorporating a larger, more diverse participant pool. These studies should include participants across different age groups, more balanced gender distribution, various ethnic and cultural backgrounds, and different occupational stress exposures. Longitudinal studies would be particularly valuable to assess the system's reliability over extended periods and validate its applicability across different stress conditions. The research should also expand to include clinical populations, particularly patients diagnosed with anxiety, depression, and other stress-related disorders, to validate the model's effectiveness in therapeutic settings. Methodologically, future studies should explore a broader range of stress-induction methods to better reflect real-world stressors and their varied impacts on physiological responses. With the rapid advancement of technology, the integration of additional physiological and psychological indicators into the stress quantification system could provide more comprehensive assessment capabilities. These technological enhancements would facilitate the system's application in various scenarios, from clinical interventions to daily life monitoring, enabling more personalized stress management recommendations.^69^

The current findings demonstrate that while PPG-derived HRV parameters effectively capture baseline stress states (R^2^ = 0.8457), the correlation decreases during active interventions (R^2^ = 0.3006 for Stroop phases, R^2^ = 0.0696 for relaxation). This pattern suggests that stress responses during these dynamic phases involve complex physiological mechanisms that might be better captured through multimodal monitoring. Electrodermal activity (EDA) would be particularly valuable as it directly reflects sympathetic nervous system activation through skin conductance changes, complementing the autonomic balance information provided by HRV.^70,71^ Facial expression analysis could add another dimension by capturing behavioral stress manifestations, especially relevant during cognitive tasks like the Stroop test. For machine learning architecture improvements, temporal pattern recognition would be crucial for capturing stress dynamics. Long Short-Term Memory (LSTM) networks would be particularly suitable for processing the temporal sequences of physiological signals, as they can capture both short-term stress responses and long-term stress patterns.^72^ The LSTM's ability to maintain information about previous states would be especially valuable during transition periods, such as between stress induction and recovery phases. Hidden Markov Models (HMMs) could complement this approach by modeling the discrete state transitions in stress levels, potentially improving the detection of subtle changes during relaxation phases. The integration of these additional modalities and advanced machine learning techniques would require careful consideration of practical implementation aspects.^73^ The system's sampling frequency would need to be optimized to handle multiple data streams while maintaining real-time processing capability.^74^ Additionally, the model architecture would need to account for different temporal scales across modalities—for instance, EDA typically shows slower response patterns compared to HRV parameters. A hierarchical implementation approach could begin with individual modality processors handling signal-specific preprocessing and feature extraction. This would be followed by a temporal integration layer using LSTM for processing the time-series aspects of each modality. Subsequently, a fusion layer would combine features across modalities while accounting for their temporal relationships. The final classification layer, potentially incorporating HMM, would produce refined stress state estimates. This multimodal approach, combined with advanced temporal pattern recognition, could particularly improve stress quantification during dynamic phases where the current system shows lower correlations with psychological measures. The development of such an enhanced system would represent a significant step toward more comprehensive stress monitoring in both clinical and real-world settings.

The use of the Stroop test as the primary stress-induction method in this study merits detailed discussion. The Stroop test was specifically chosen for several key advantages in stress research. First, it provides a standardized and reproducible cognitive stressor, allowing for precise control over stress intensity through timing constraints and additional auditory interference. The test's well-documented ability to induce measurable physiological stress responses makes it particularly suitable for validating stress detection systems. Research has shown that the Stroop test reliably activates the sympathetic nervous system, leading to quantifiable changes in cardiovascular parameters, including heart rate variability.^75^ Additionally, the test's dual-task nature requiring participants to suppress automatic word reading while naming colors creates a cognitive conflict that closely mirrors real-world stress scenarios where individuals must manage competing demands. Unlike social or physical stressors, the Stroop test's cognitive nature makes it especially relevant for studying stress in modern workplace environments where cognitive overload is increasingly common. However, it is important to acknowledge that individual variability in cognitive processing and stress resilience may influence responses to the Stroop test. Future studies could benefit from incorporating multiple stress-induction methods, such as the Trier Social Stress Test or arithmetic challenges, to provide a more comprehensive understanding of stress responses across different types of stressors.^24,76,77^

The findings of this study also open up promising avenues for predicting circulatory pathologies, particularly those affecting cerebral blood circulation and stroke risk. The relationship between HRV parameters and autonomic nervous system function suggests potential applications in early detection of cardiovascular and cerebrovascular risks.^78^ The results demonstrate that psychological stress significantly impacts various HRV parameters, including frequency-domain measures like LF/HF ratio and time-domain measures like SDNN, which are known indicators of autonomic nervous system balance.^79^ Since chronic autonomic dysfunction is a recognized risk factor for cerebrovascular events, monitoring these parameters over time could provide valuable insights into stroke risk assessment.^80^ The strong correlation between the stress quantification model and psychological assessments (DASS-21-C) suggests that this system could be particularly valuable for monitoring individuals with increased risk of stress-related circulatory pathologies. The observed sex differences in stress recovery patterns might also have implications for personalized risk assessment, as these differences could influence the development and progression of cerebrovascular conditions. For instance, the finding that females show slower recovery from severe stress could indicate a need for more targeted monitoring in this population. Future research could focus on longitudinal studies to establish the predictive value of these HRV parameters for cerebrovascular events. By tracking changes in HRV patterns over extended periods and correlating them with clinical outcomes, researchers could potentially develop early warning systems for stroke risk. This could be particularly valuable for identifying subtle changes that precede transient ischemic attacks (TIAs) or mini-strokes, which often serve as warning signs for more severe cerebrovascular events. The wearable nature of the device makes it well-suited for such long-term monitoring applications. Additional research directions could include integrating the HRV monitoring system with other cardiovascular risk markers, such as blood pressure variability patterns, inflammatory markers, and carotid artery ultrasound data. This multi-modal approach could provide a more comprehensive risk assessment tool for circulatory pathologies. Furthermore, the real-time nature of the system could enable the detection of acute stress-induced changes in cardiovascular function, potentially identifying critical periods when individuals might be at increased risk for cerebrovascular events. The current study addresses several important ethical considerations in stress monitoring and data management. Data collection in this phase was conducted in a controlled laboratory environment with strict protocols for participant privacy. All physiological data were de-identified during storage, with participant information separated from the physiological measurements. Looking toward clinical applications, the system is designed to integrate with existing hospital information systems, where data security is maintained through established institutional protocols. The data access would be restricted to authorized health care providers, with all information stored locally within the hospital's secure infrastructure. This approach ensures that sensitive physiological data remain protected under the hospital's comprehensive security measures. Regarding real-time stress feedback, the system is designed to provide quantitative stress indicators specifically to health care professionals, physicians and psychologists as a supplementary tool for treatment monitoring and adjustment. This professional-mediated approach helps ensure that stress measurements are interpreted within proper clinical context and used appropriately to inform treatment decisions. The stress quantification data serve as objective reference points for healthcare providers to track treatment progress and adjust interventions as needed, rather than as direct feedback to patients.

The current implementation of the device utilizes a Type-C connection to interface with a computer, which eliminates battery life constraints and ensures stable signal acquisition in clinical settings. This design choice aligns with the primary intended use case in hospital environments, where continuous power supply and reliable data transmission are essential for accurate stress monitoring during therapy sessions. However, we acknowledge that future applications may require more flexible deployment options, particularly for home-based monitoring. Future development plans include adapting the system for wireless wearable applications through integration with existing smartwatch-type PPG devices. This integration would address several key considerations for long-term monitoring: user compliance would likely improve through the familiarity and convenience of watch-type devices, signal drift could be managed through regular calibration protocols, and communication stability could be maintained through established wireless protocols. The stress quantification algorithms developed in this study could be optimized for implementation on these wearable platforms, potentially enabling continuous stress monitoring in daily life settings while maintaining measurement accuracy. Such adaptations would expand the system's applicability beyond clinical environments while preserving its core capabilities for reliable stress assessment. This study successfully developed and validated a wearable stress assessment system based on PPG for noninvasive physiological signal measurement and individual stress level quantification. The results demonstrate that PPG signals can effectively replace traditional ECG signals for calculating HRV parameters and that the developed HRV–stress model can accurately assess participants' stress states on the basis of HRV fluctuations. While normative HRV databases exist, they focus primarily on general physiological states rather than stress-induced HRV variations. Therefore, this study established a stress model via machine learning, incorporating data collected during specific stress-inducing tasks such as the Stroop test to better capture acute stress responses. A series of experiments confirmed the system's effectiveness under various stress conditions, demonstrating that mindful breathing can effectively alleviate stress. Previous studies also established a strong correlation between HRV parameters (LF nu, LF/HF) and depression.^81^ In the future, the proposed system could be applied in clinical settings to monitor patients with depression over extended periods, providing healthcare professionals with physiological data to assess patients' stress levels and recovery status. This system also has potential for application in psychological therapy, offering therapists valuable real-time indicators of stress. By monitoring these stress indicators, therapists can adjust treatment plans according to changes in a patient's stress levels, thereby improving therapeutic outcomes. With modern advancements, psychological therapy is no longer confined to in-person sessions; remote therapy, conducted via video conferencing, is becoming increasingly common. However, it can be challenging for therapists to assess a patient's condition fully during remote therapy sessions. In traditional face-to-face clinical settings, therapists rely on verbal cues, such as tone and speech rates, as well as nonverbal cues, including body language and facial expressions, to evaluate stress levels.^82^ These cues are often more difficult to observe in remote therapy.^83^ The proposed system provides an objective method for quantifying patient stress during remote therapy, allowing therapists to track stress variations and adjust their strategies in real time. For example, if a patient feels more anxious or stressed during a session, the therapist could intervene immediately by guiding the patient through deep breathing or relaxation techniques. These real-time interventions could greatly improve the effectiveness of remote psychological therapy.

Future development of the stress monitoring system could incorporate additional noninvasive physiological indicators to enhance measurement accuracy. Electroencephalography (EEG) integration could provide valuable neural correlates of stress responses through analysis of frontal alpha asymmetry and theta band power variations. These measurements could offer insight into cognitive load and emotional processing during stress responses, with millisecond-level temporal resolution enabling precise tracking of rapid stress dynamics. The development of portable, dry-electrode EEG systems makes this integration increasingly feasible for real-world applications. Electrodermal activity (EDA) measurements would provide direct insight into sympathetic nervous system activation through changes in skin conductance, offering complementary information to HRV parameters. EDA responses are particularly valuable for detecting acute stress reactions and could improve system performance during active stress phases where current correlations are moderate. Thermal imaging presents another promising modality through monitoring of facial temperature variations, particularly in the periorbital and supraorbital regions. These temperature changes reflect autonomic nervous system activity and could provide non-contact measurement capability, potentially improving user comfort during long-term monitoring. The combination of thermal imaging with existing PPG measurements could enhance the detection of stress-induced cardiovascular changes, particularly during dynamic stress states. The integration of these additional physiological indicators would require careful consideration of sampling rates and signal processing requirements while maintaining the system's wearability and practical utility in clinical settings. Such enhancements could particularly benefit remote therapy applications, where multiple physiological indicators could provide therapists with a more comprehensive view of patient stress states during virtual sessions. The challenge lies in developing efficient algorithms for real-time processing of these multimodal signals while maintaining system portability and ease of use.

## IV. CONCLUSION

In this study, a wearable device based on photoplethysmography (PPG) technology was developed and validated, enabling noninvasive measurement of physiological signals and quantification of individual stress levels. The feasibility of using PPG signals was confirmed through linear regression analysis between PPG and ECG signals for RR intervals, which revealed a strong positive correlation (correlation coefficient = 0.9918, R-squared = 0.9837), demonstrating that PPG signals can effectively replace ECG signals in calculating HRV parameters. Further validation of the self-developed wearable device was performed by comparing its PPG signal RR interval analysis with that of the commercial MAX86150 device. The results indicated strong correlations in terms of RR interval linear regression (correlation coefficient = 0.9166, R-squared = 0.8401), overlaid charts (correlation coefficient = 0.8965), and frequency spectrum analysis (correlation coefficient = 0.7817), confirming the accuracy and reliability of the developed device in measuring PPG signals and calculating HRV. After the HRV–stress model was trained, stress experiments were conducted to validate the model. The Stroop test, with and without interference, was used to simulate mild and severe stress, respectively, while mindful breathing was used during the relaxation phase. The results showed that the stress scores calculated by the model for each experimental phase aligned with expectations, with significant increases in stress levels during the Stroop phase (*p* < 0.05). In addition to the use of the HRV–stress model for defining stress, the DASS-21-C psychological questionnaire was employed to quantify stress levels. A linear regression analysis of the stress scores from both methods revealed a strong positive correlation during the baseline (correlation coefficient = 0.92) and recovery phases (correlation coefficient = 0.81). This high correlation, particularly in the absence of external interference, suggests that the HRV–stress model is aligned with traditional psychological assessments, adding psychological significance to the stress measurements defined by the model. This study also examined physiological differences in stress responses between sexes. Males demonstrated faster recovery from acute stress, with HRV parameters such as SDNN, RMSSD, and LF/HF indicating significant recovery trends. These findings suggest that males are more efficient at restoring the balance between the sympathetic and parasympathetic nervous systems after severe stress. In contrast, females displayed weaker self-regulation following severe stress, with slower recovery of parameters such as RMSSD, HF, and LF/HF, indicating that sex differences in stress hormone regulation (e.g., cortisol) and endocrine factors (e.g., estrogen) may contribute to these disparities. Through these validations, the study demonstrated that the developed model can effectively quantify stress. In future clinical applications, the system could assist doctors in diagnostic decision-making and enable psychologists to monitor patients' stress levels in real time during therapy. This would facilitate more informed treatment planning and position the system as a valuable tool for monitoring stress in patients with mental health disorders, such as anxiety and depression. Considering the close relationship between stress and the progression of these conditions, the wearable device and HRV model developed in this study could provide real-time, objective stress data to help healthcare professionals formulate effective treatment strategies throughout the therapeutic process.

## V. METHODS

### A. Stress quantification process

**FIG. 6.**
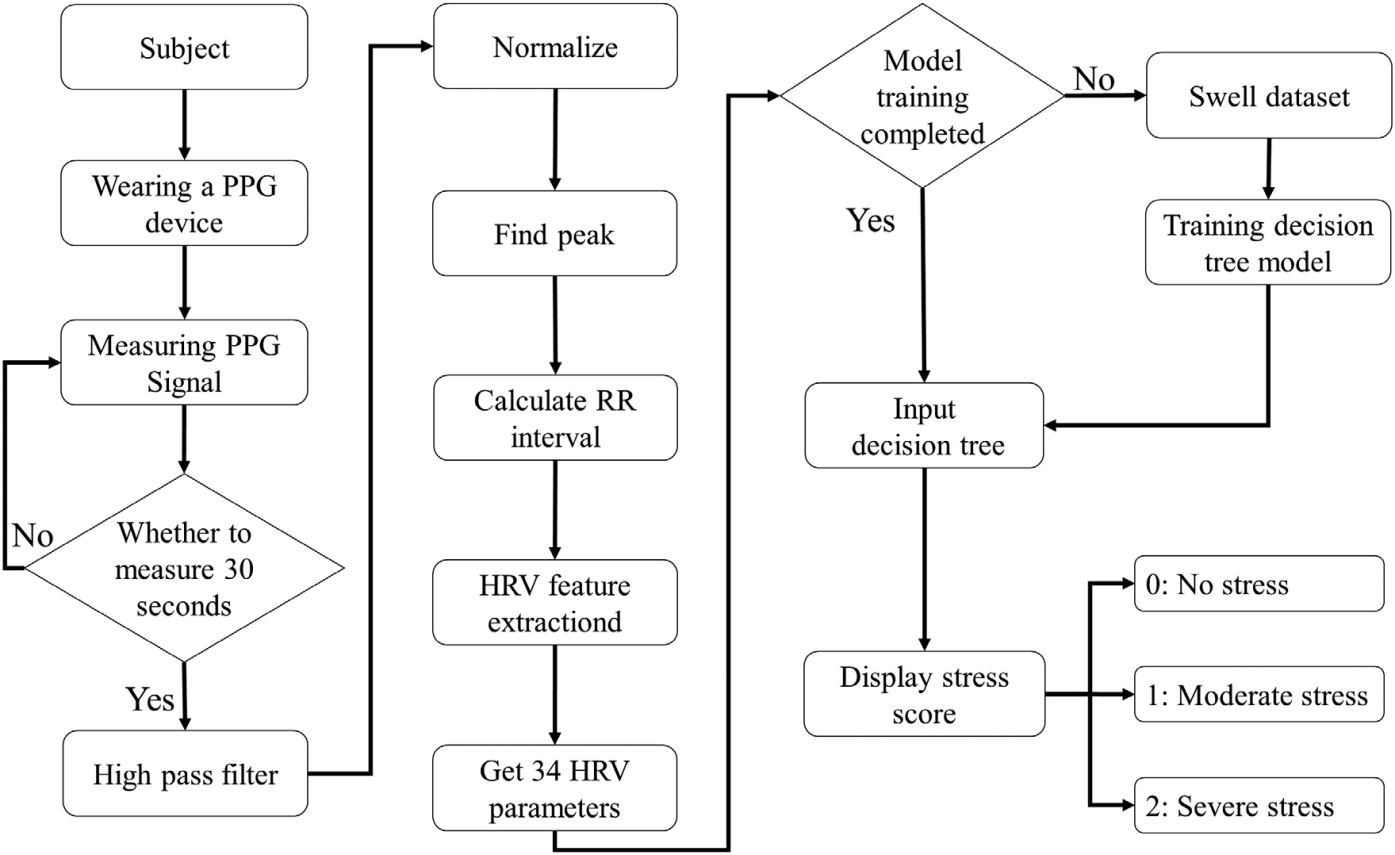
Stress measurement flow chart. The process begins with the user attaching the PPG measurement device to their index finger, after which the PPG signal is measured. Following 30 s of measurement, signal processing is performed. Initially, a high-pass filter with a cutoff frequency of 0.5 Hz is applied to remove DC noise. The next step is normalization, where the PPG signal values are compressed to between 0 and 1. After normalization, peak detection is conducted with a threshold of 0.5 to identify the peaks in the 30-s PPG signal. The RR interval values are then calculated by subtracting the adjacent peak values. Various HRV formulas are subsequently used to extract 34 HRV parameter features, which include both time-domain and frequency-domain features. These 34 HRV parameters are then input into the HRV–stress decision tree model to assess the stress state. If the model has not been fully trained, the Swell dataset is used as a training set to train the model. Once the training is complete, the 34 HRV parameters are input, and the final output is a stress state score. This score ranges from 0 to 2, representing no stress (0), mild stress (1), or severe stress (2).

Figure 6 illustrates the process of quantifying stress levels via PPG. First, the participants wore the PPG device, and after 30 s of measurement, the system processed the signals.^84^ The raw PPG signals were filtered via a high-pass Butterworth filter with a 0.5 Hz cutoff frequency to eliminate direct current noise. The signal values were then normalized to between −1 and 1 by dividing the signal within the 30-s window by its maximum value. Next, the system detected signal peaks and calculated RR intervals, from which HRV parameters were derived. A total of 34 HRV parameters were computed: 11 time-domain parameters, 8 relative time-domain parameters, 11 frequency-domain parameters, and 4 nonlinear parameters, including SD1, SD2, sample entropy (SampEn), and Higuchi's fractal dimension (Higuchi).

The time-domain parameters included the mean RR interval (mean RR), median RR interval (median RR), standard deviation of normal-to-normal intervals (SDNN), root mean square of successive differences (RMSSD), standard deviation of differences between adjacent RR intervals (SDSD), the SDNN/RMSSD ratio, heart rate (HR), pNN25, pNN50, kurtosis (KURT), and skewness (SKEW).^85^ The relative time-domain parameters were computed by first normalizing the RR intervals to reduce individual variability. Parameters such as the mean RR, median RR, SDNN, RMSSD, SDSD, SDNN/RMSSD, KURT, and SKEW were recalculated from these normalized values.^86^ In the frequency domain, spectral power density analysis of the RR intervals was conducted to compute power in the very low frequency (VLF, 0.003–0.04 Hz) range, low frequency (LF, 0.04–0.15 Hz) range, and high frequency (HF, 0.15–0.4 Hz) range, as well as the total HRV power spectrum (TF), LF/HF ratio, and HF/LF ratio.^87^ Nonlinear parameters such as SD1, SD2, and SampEn were used to quantify the complexity of the time series, and Higuchi's fractal dimension was used to measure the fractal nature of the RR intervals.^88,89^

These 34 HRV parameters were then input into the HRV–stress decision tree model to evaluate the participant's stress level. Before the model was trained, the publicly available Swell dataset, which contains stress experiment data, was used to train and validate the model.^85,90^ Once trained, the model classified the participants' stress states into one of three categories: no stress (0), moderate stress (1), and severe stress (2). These classifications can provide valuable insights for psychologists and medical professionals, aiding in the development of more effective treatment plans.

### B. Wearable device mechanism diagram and PPG measurement system architecture diagram

**FIG. 7.**
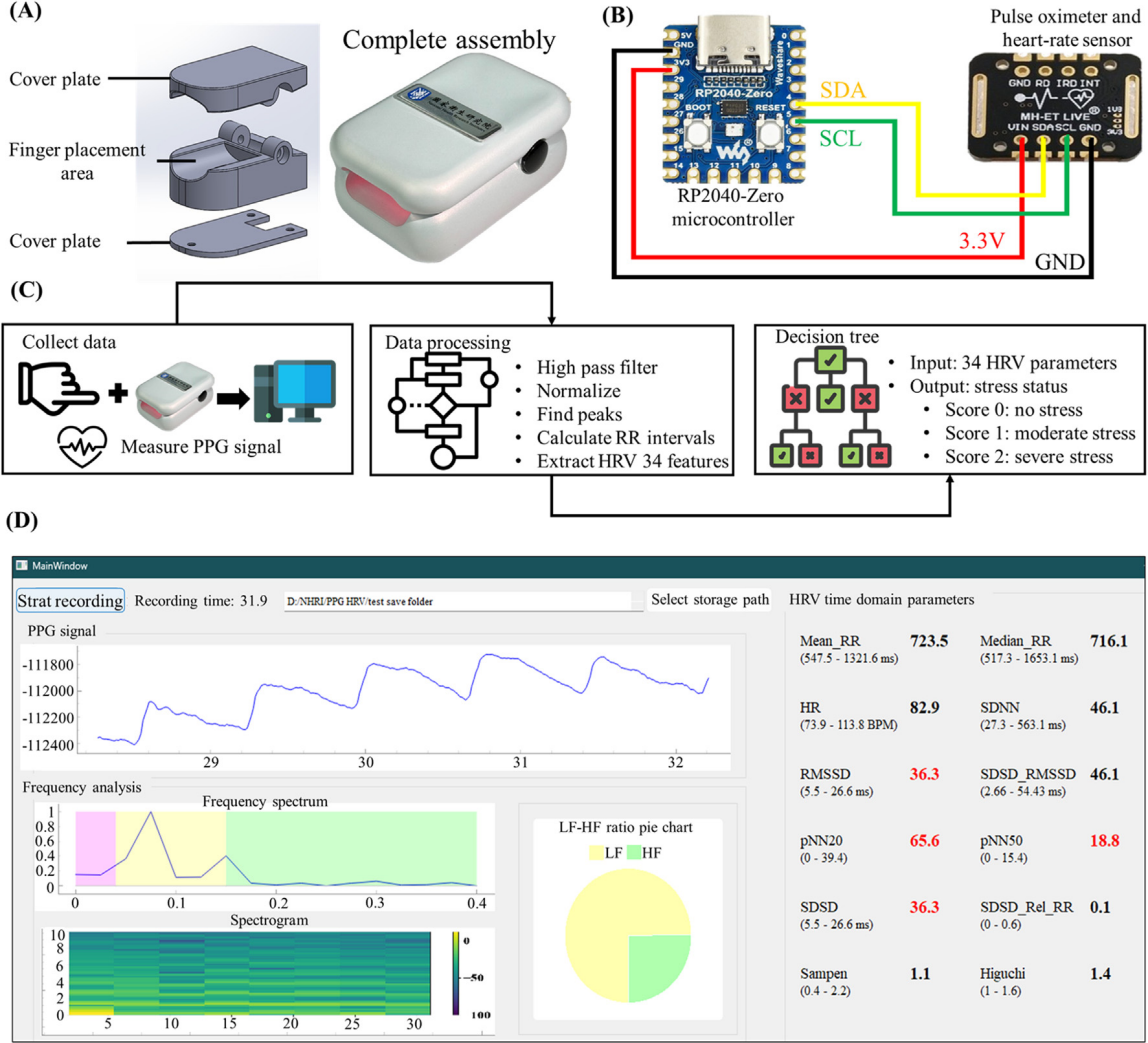
Sensor mechanism diagram and device system architecture diagram. (a) Shell structure design diagram: The mechanism consists of three main components: the top cover, the main body, and the bottom cover. The sensor is placed inside the main body and secured with the bottom cover to reduce excessive noise caused by movement. Once the top cover is locked in place, the device is fully assembled. A groove between the top cover and the main body ensures a better fit for the finger, minimizing signal noise. The actual product is shown on the right side of the diagram. (b) Signal measurement unit: This unit includes a microcontroller and a blood oxygen measurement module. A Raspberry Waveshare RP2040-zero microcontroller is used, and it communicates with the blood oxygen measurement module via I^2^C. The blood oxygen module transmits photodetector signals to the microcontroller at a sampling rate of 100 Hz. After receiving the signals, the microcontroller instantly transmits them to a computer, where the PPG signal is displayed in the GUI. (c) System architecture diagram: The device is designed as a finger-clip wearable device. Once connected to the computer, it can be operated through a custom-designed GUI. When the device is clipped onto the index finger and the measurement is initiated in the GUI, the real-time PPG signal is displayed. After 30 s of measurement, the raw signal undergoes several processing steps, including high-pass filtering, normalization, peak detection, RR interval calculation, and HRV parameter extraction. After the 34 HRV parameters are extracted, they are input into the decision tree model, which generates a stress score. Scores of 0, 1, and 2 represent no stress, mild stress, and severe stress, respectively. (d) Signal measurement interface: Upon pressing the start button, the real-time PPG signal is displayed in the PPG signal area of the interface. Every 30 s, the HRV parameters are automatically calculated and displayed on the right side of the interface. Additionally, the frequency spectrum, time–frequency diagram, and LF/HF pie chart of the PPG signal are shown at the bottom of the interface. The system also automatically saves the raw PPG data and the calculated HRV parameters in a CSV file for further analysis. Multimedia available online.10.1063/5.0256590.2

In this study, a wearable stress detection device was designed and implemented, as illustrated in Fig. 7. The system includes the complete process from hardware assembly to data collection, processing, and result display, with the aim of providing real-time stress monitoring and assessment. Figure 7(a) shows a diagram of the wearable device, which includes upper and lower covers, both of which are 3D printed. Inside, there is a designated area for finger placement to secure the finger, enabling accurate PPG signal measurement. A gap is created in the internal hardware to accommodate the microprocessor and sensors. When the user wears the device, the sensor measures blood volume changes at the fingertip and transmits the signals to the microprocessor. Figure 7(b) depicts the sensor composition, including a microprocessor and a pulse oximetry sensor module. The microprocessor is an RP2024-Zero (Raspberry Pi), and a MAX30102 pulse oximetry sensor module (Analog Devices) is used for signal acquisition. Communication between the sensor and the microprocessor occurs via the I^2^C protocol, a commonly used communication method in integrated circuits. I^2^C consists of two main lines: a serial data line (SDA) for data transmission and a serial clock line (SCL) for synchronization and data transmission control, ensuring both accuracy and stability in data transfer.^91,92^ Figure 7(c) shows the architecture of the PPG system. When the user wears the device, PPG signal measurement begins, and real-time signals are transmitted to a computer for processing. The system applies a high-pass filter to remove DC signal noise, normalizes the data, and detects signal peaks. Once the peaks are identified, RR intervals are calculated from the PPG signal, and 34 HRV parameters are derived. These parameters are input into the decision tree model, which assigns a stress score. The user's stress state is categorized into one of three levels: 0 for no stress, 1 for moderate stress, and 2 for severe stress.

Figure 7(d) (Multimedia view) shows the graphical user interface for measuring PPG signals. Users can set the data storage path and manually press “start recording.” Once the device is activated, the PPG waveform is displayed in real time. Every 30 s, the HRV parameters are recalculated, and the interface automatically updates the spectrum plot, time-frequency plot, LF/HF pie chart, and time-domain HRV parameters. This interface simplifies operation and data interpretation, enhancing both practicality and the user experience. The wearable stress detection device and PPG measurement system developed in this study provide real-time stress monitoring and assessment through integrated hardware and software. This system has potential applications in personal health monitoring and stress management.^93^

### C. Verification of PPG signal feasibility

In previous studies, HRV parameter calculations have relied primarily on electrocardiogram (ECG) signals. This study aims to evaluate the feasibility of using PPG signals as a substitute for ECG signals in HRV parameter calculations. The first step of the experiment is to verify whether PPG signals can reliably serve as a standard for calculating HRV parameters. Traditionally, HRV parameters are derived by detecting R-wave peaks in ECG signals to compute RR intervals, which are then used for HRV calculations over a specific period. In this study, the MAX86150 (Maxim Integrated MAX86150 Evaluation Kit, Analog Devices), which is capable of simultaneously measuring both ECG and PPG signals, was used as the verification tool.^94^ In the experiment, each participant simultaneously measured ECG and PPG signals for five minutes. Peaks from both signals were extracted to calculate RR intervals, and a linear comparison was conducted to assess the feasibility of using PPG signals as a substitute for ECG signals in HRV parameter calculation.

Given that the PPG sensor used in the wearable device developed in this study (MAX30102 pulse oximetry module) differs from the sensor used in the MAX86150, a secondary validation experiment was needed to ensure consistency between the PPG signals measured by the two sensors. In this experiment, a participant simultaneously wore both the wearable device developed in this study and the MAX86150 device to measure PPG signals continuously for five minutes. A correlation analysis was performed on the two sets of PPG signals in the time domain. Additionally, the signals underwent the fast Fourier transform (FFT) to convert them from the time domain to the frequency domain, and the frequency domain signals were compared. The peaks of both PPG signals were also detected to calculate RR intervals, followed by a linear comparison to further verify the feasibility of using the wearable device developed in this study for HRV parameter calculation. Through these validation experiments, this study aimed to demonstrate the feasibility of using PPG signals as a reliable standard for calculating HRV parameters. Additionally, the experiments confirmed the accuracy and reliability of the wearable device developed in this study for practical applications in stress monitoring.

### D. Stress model construction

The stress model in this study aims to predict the stress states of participants via a decision tree approach. The model is trained and validated using the Swell Knowledge Work Dataset (SWELL-KW), which was developed by Koldijk and made available by the Institute for Computing and Data Sciences at Radboud University in Nijmegen, the Netherlands. This comprehensive dataset was specifically designed to study stress and user modeling in knowledge workers within office environments, making it particularly relevant for workplace stress analysis applications. The open dataset consists of data from 25 participants (17 males and 8 females, with an average age of 25 ± 3.25 y). Similarly, this study included 25 participants (15 males and 10 females, with an average age of 24.9 ± 2.9 y). The close alignment in participant demographics, including sample size, sex distribution, and age range, between the two datasets makes the Swell dataset particularly suitable as a basis for model construction and validation. The dataset was collected in a controlled office environment simulation designed to examine the effects of work-related stress on participants' physiological and psychological states. The data collection protocol involved three distinct working conditions: neutral, time pressure, and interruptions, each lasting approximately 45 min. During these sessions, participants performed knowledge work tasks such as writing reports and making presentations while experiencing different stressors typical of office environments. The dataset's strength lies in its multimodal approach to stress monitoring. The collected data encompass physiological measurements, including ECG signals and electrodermal activity, alongside behavioral indicators from computer interaction logs and application usage patterns. Physical responses were tracked through facial expressions and body posture monitoring, while subjective assessments included self-reported measures of task load, mental effort, emotions, and perceived stress levels. Environmental factors such as ambient noise levels and workspace conditions were also recorded throughout the sessions. Participants' stress levels were systematically manipulated through controlled stressors, and their responses were carefully documented using validated psychological and physiological measures. This comprehensive approach to data collection, combined with careful experimental design and thorough documentation, makes the Swell dataset particularly valuable for developing and validating stress detection models. The stress model in this study aims to predict the stress states of participants via a decision tree approach. The model is trained and validated via the Swell dataset, which is provided by Koldijk and made available by the Institute for Computing and Data Sciences at Radboud University in Nijmegen, the Netherlands. This open dataset consists of data from 25 participants, 17 males and 8 females, with an average age of 25 ± 3.25 years. Similarly, this study included 25 participants, 15 males and 10 females, with an average age of 24.9 ± 2.9 years. The similarities in the participant numbers, sex distributions, and age ranges between the two datasets make the Swell dataset suitable as a basis for model construction. An office environment was simulated to collect the dataset; the goal was to examine the effects of work-related stress on participants' physiological and psychological states. Participants were exposed to varying levels of work-related stress, and data were collected regarding multiple modalities, including computer logs, facial expressions, body posture, ECG signals, and EDA, as well as subjective data such as task load, mental effort, emotions, and perceived stress.^95^

For model construction, a decision tree algorithm is chosen because of its simplicity and interpretability.^46^ The Swell dataset is presplit into training and testing sets, where the training set is used to train the model and the test set is reserved for validation. HRV parameters are extracted from the training set, including time-domain, frequency-domain, and nonlinear parameters, totaling 34 features. These features serve as input variables for the decision tree, and the target variable is the participant's stress state (no stress, moderate stress, or severe stress). The decision tree recursively splits the data into smaller subsets, maximizing the homogeneity within each subset on the basis of feature thresholds, until a stopping criterion is met.

To optimize the model's performance, hyperparameter tuning is carried out. The key hyperparameters include the maximum depth of the tree (Max Depth), which is set to 10 to prevent overfitting; the minimum number of samples required to split an internal node (Min Samples Split), which is set to 10; and the minimum number of samples required at each leaf node (Min Samples Leaf), which is set to 5. After training, the model's performance is evaluated on the test set via metrics such as accuracy, precision, recall, and F1 score. Accuracy reflects the proportion of correct predictions, precision assesses the accuracy within each class, recall measures the model's ability to correctly identify all relevant instances, and the F1 score provides a balanced measure of precision and recall.^96^ Once trained, the decision tree model classifies participants' stress states on the basis of the HRV parameters into three categories: no stress (0), moderate stress (1), and severe stress (2).

Although other machine learning architectures, such as random forest (RF) and convolutional neural networks (CNNs), have stronger nonlinear fitting capabilities, the decision tree was chosen because of its simpler structure and higher execution efficiency, which make it ideal for small datasets. In cases where the dataset size is limited, the use of complex models such as RF or CNNs can lead to overfitting, thereby reducing generalizability to unseen data.^97,98^ Additionally, decision trees have faster execution times and lower hardware requirements, enabling real-time feedback and practical implementation. Therefore, the decision tree is adopted as the primary approach in this study.

### E. Self-report scale: Depression, Anxiety, and Stress Scale, Chinese version (DASS-21-C)

The Depression, Anxiety, and Stress Scale is a self-reported scale for assessing depression, anxiety and stress-related symptoms.^99^ The DASS-21, a short version of the original 42-item scale, consists of 21 items divided into three subscales (depression, anxiety, and stress), each containing seven items.^100^ The depression subscale evaluates symptoms such as dejection, loss of enjoyment, and hopelessness. The anxiety subscale measures physiological responses and sensations related to anxiety, including autonomic arousal, situational anxiety, and subjective experiences of anxiety. The stress subscale assesses chronic nonspecific arousal, including difficulty relaxing, overreaction, and impatience.

The Depression, Anxiety, and Stress Scale, Chinese version (DASS-21-C) is a translated version of the original DASS-21. To evaluate the structural validity of the DASS-21-C, confirmatory factor analysis (CFA) was conducted. CFA commonly uses three key indices to assess model fit: the comparative fit index (CFI), Tucker–Lewis index (TLI), and root mean square error of approximation (RMSEA).^101,102^ The CFI compares the research model against a null model, with values ranging from 0 to 1, where a value closer to 1 indicates better fit. For the DASS-21-C, the CFI value was 0.91, suggesting a good model fit. The TLI, another fit index, adjusts for model complexity, with values ranging from 0 to 1; a TLI of 0.90 for the DASS-21-C indicates that the model meets recognized standards. The RMSEA measures the discrepancy between the model and the data, with lower values indicating better fit; the RMSEA for the DASS-21-C is 0.07, meeting the criteria for reasonable fit. Taken together, the CFI, TLI, and RMSEA indices suggest that the DASS-21-C has good structural validity, making it a reliable tool for assessing participants' anxiety, stress, and depression states in this study.^103,104^

### F. Stress induction method

The experiment was conducted in a 4.3 × 3.7 square meter room, with ambient noise levels kept below 40 decibels (dB). Only one participant and one researcher were present in the room to minimize environmental variables and ensure the accuracy and consistency of the results. In this study, the Stroop test was used as the primary method of stress induction. The Stroop test is a well-established psychological tool that is effective for studying cognitive interference and selective attention. It was first introduced by the American psychologist John Ridley Stroop in 1935, and it induces psychological stress by testing participants' response abilities via cognitive conflicts.^76^ In a typical Stroop test, participants are presented with words that have incongruent meanings and font colors, such as the word “red” displayed in green. The participants must ignore the word's meaning and report the font color as quickly and accurately as possible. This task creates cognitive conflict, as the brain processes the word's meaning automatically, requiring additional effort to focus on the font color. As a result, response times are longer and error rates are higher than they would be in a case without cognitive conflict, which induces stress.^77^

To induce further stress, a custom Stroop test interface was designed with two stress conditions. The first condition involved a five-minute Stroop test, where participants were required to respond within two seconds, adding time pressure to the cognitive task. The second condition introduced auditory interference on top of the first task. When a word was shown, the system played audio of a person reading the word, which was mismatched with the font color. For example, if the word red is displayed in green, the system plays audio of someone saying red. This auditory interference further increased the cognitive load, requiring participants to overcome both sensory distractions and time pressure, thus inducing greater psychological stress. Through this design, the Stroop test effectively induced psychological stress in participants under varying stress conditions. By measuring physiological signals, this study quantified and compared participants' stress responses, providing data to support further research.

### G. Experimental procedure for validating the feasibility of the stress model

In this study, a series of experimental steps were designed to validate the feasibility of the stress model, as illustrated in Fig. 5(a). First, participants completed the DASS-21-C questionnaire, which quantified their anxiety, depression, and stress levels. This study focused on comparing participants' stress scores with decision tree model results to establish a correlation between psychological stress, as defined in psychology, and stress, as quantified through engineering methods. The experiment began with participants sitting quietly for five minutes while their PPG signals were measured, providing baseline data (Rest 1). Next, the participants underwent a five-minute Stroop test (Stroop 1), during which their PPG signals were recorded. The Stroop test is widely recognized for inducing short-term stress, and data from this phase were classified as mild stress. Following the initial stress phase, the participants sat quietly for ten minutes (Rest 2) so that HRV recovery under mild stress conditions could be observed. The participants then proceeded to a second five-minute Stroop test (Stroop 2), which included auditory interference to increase stress intensity; this phase was therefore defined as high stress. Afterward, the participants entered another ten-minute quiet period (Rest 3) to measure HRV recovery under high-stress conditions. Finally, the participants watched a relaxation video for ten minutes (Relax), during which their PPG signals were recorded to assess changes in HRV parameters in response to external relaxation stimuli. Two relaxation video options were provided: ocean scenery and mindfulness breathing. Initially, five participants compared the relaxation effects of the two videos to determine the more effective option for use in subsequent experiments.

Figure 5(b) shows the actual experimental setup, where participants face a screen providing visual stimuli for stress or relaxation, and their left index finger is equipped with a fingertip-worn device to measure PPG signals. The HRV parameters are calculated every 30 s, and the raw PPG data and HRV parameters are automatically stored in separate CSV files. These HRV parameters are then input into the decision tree model to analyze participants' stress trends throughout the experiment. This design allows for the effective validation of the stress model by comparing psychological stress indices with physiological data and verifying the model's accuracy and reliability. There were a total of 25 participants in this experiment, with an average age of 24.8 ± 2.9 years, including 15 males and 10 females.

## SUPPLEMENTARY MATERIAL

See the supplementary material for details supplementary material 1 (Ocean video) and supplementary material 2 (Mindful breathing video).

## Data Availability

The data that support the findings of this study are available from the corresponding author upon reasonable request.
